# Global evaluation of taxonomic relationships and admixture within the *Culex pipiens* complex of mosquitoes

**DOI:** 10.1186/s13071-020-3879-8

**Published:** 2020-01-08

**Authors:** Matthew L. Aardema, Bridgett M. vonHoldt, Megan L. Fritz, Steven R. Davis

**Affiliations:** 1 Department of Biology, Montclair State University, Montclair, NJ USA; 2 Sackler Institute for Comparative Genomics, American Museum of Natural History, New York, NY USA; 3 Ecology and Evolutionary Biology, Princeton University, Princeton, NJ USA; 4 Department of Entomology, University of Maryland, College Park, MD USA; 5 Division of Invertebrate Zoology, American Museum of Natural History, New York, NY USA

**Keywords:** Mosquito, Culicidae, Disease vector, Population structure, Species complex, Genetic exchange

## Abstract

**Background:**

Within the *Culex pipiens* mosquito complex, there are six contemporarily recognized taxa: *Cx*. *quinquefasciatus*, *Cx*. *pipiens* f. *pipiens*, *Cx*. *pipiens* f. *molestus*, *Cx*. *pipiens pallens*, *Cx*. *australicus* and *Cx*. *globocoxitus*. Many phylogenetic aspects within this complex have eluded resolution, such as the relationship of the two Australian endemic taxa to the other four members, as well as the evolutionary origins and taxonomic status of *Cx*. *pipiens pallens* and *Cx*. *pipiens* f. *molestus*. Ultimately, insights into lineage relationships within the complex will facilitate a better understanding of differential disease transmission by these mosquitoes. To this end, we have combined publicly available data with our own sequencing efforts to examine these questions.

**Results:**

We found that the two Australian endemic complex members, *Cx*. *australicus* and *Cx*. *globocoxitus*, comprise a monophyletic group, are genetically distinct, and are most closely related to the cosmopolitan *Cx*. *quinquefasciatus*. Our results also show that *Cx*. *pipiens pallens* is genetically distinct, but may have arisen from past hybridization. Lastly, we observed complicated patterns of genetic differentiation within and between *Cx*. *pipiens* f. *pipiens* and *Cx*. *pipiens* f. *molestus*.

**Conclusions:**

Two Australian endemic *Culex* taxa, *Cx*. *australicus* and *Cx*. *globocoxitus*, belong within the *Cx. pipiens* complex, but have a relatively older evolutionary origin. They likely diverged from *Cx*. *quinquefasciatus* after its colonization of Australia. The taxon *Cx*. *pipiens pallens* is a distinct evolutionary entity that likely arose from past hybridization between *Cx*. *quinquefasciatus* and *Cx*. *pipiens* f. *pipiens*/*Cx. pipiens* f. *molestus*. Our results do not suggest it derives from ongoing hybridization. Finally, genetic differentiation within the *Cx*. *pipiens* f. *pipiens* and *Cx*. *pipiens* f. *molestus* samples suggests that they collectively form two separate geographic clades, one in North America and one in Europe and the Mediterranean. This may indicate that the *Cx*. *pipiens* f. *molestus* form has two distinct origins, arising from *Cx*. *pipiens* f. *pipiens* in each region. However, ongoing genetic exchange within and between these taxa have obscured their evolutionary histories, and could also explain the absence of monophyly among our samples. Overall, this work suggests many avenues that warrant further investigation.
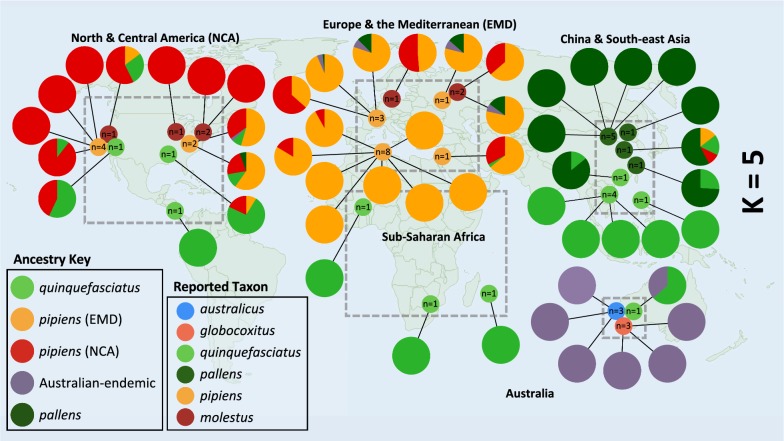

## Background

Collections of very closely related taxa present a challenging problem for evolutionary biologists and taxonomists, as they often exhibit limited morphological and genetic divergence [1]. In such cases, this lack of divergence makes confident taxonomic distinctions difficult, particularly when sampled lineages represent various stages of divergence. Incomplete lineage sorting and genetic exchange between seemingly distinct species further complicates the tasks of categorizing discrete groups and analyzing their evolutionary origins [2]. However, such challenging groups of taxa also present fascinating opportunities to explore the very processes that generate taxonomic and ecological diversity [3]. Furthermore, when closely related taxa differ in physiology, behavior, and/or ecology that affect their ability to vector human pathogens, the need for a clear understanding of the relationships between species and populations is critical for understanding their evolutionary history, evaluating potential disease transmission cycles, and establishing control strategies [4].

The globally distributed mosquitoes of one such taxonomic collection are commonly referred to as the *Culex pipiens* species complex. Within this group are six contemporarily recognized taxa: *Culex pipiens* f. *pipiens, Cx. pipiens* f. *molestus, Cx. pipiens pallens, Cx. quinquefasciatus, Cx. australicus* and *Cx. globocoxitus* [5–7]. For the sake of simplicity and to avoid unnecessary taxonomic assumptions, for the remainder of this paper we will use each taxon’s specific epithet alone.

Many questions about the *Cx. pipiens* complex have alluded resolution. For example, the relationship of the Australian endemic members of the complex, *australicus* and *globocoxitus*, to the four other taxa in the group remains uncertain [7–9]. In the laboratory, *australicus* and *globocoxitus* will interbreed with other members of the complex [10, 11]. Probable hybrids between *globocoxitus* and *molestus* have also been collected in the field [10]. However, while crosses between *globocoxitus* males and *molestus* females in the laboratory were fertile, in the reciprocal cross females appeared nearly completely sterile and what larvae were produced failed to develop to adulthood [12]. Some authors have postulated an early divergence of *australicus* and *globocoxitus* from the rest of the complex [13, 14], but little genetic work has been done to examine this hypothesis explicitly. Other authors have discussed whether these taxa belong to the *Cx. pipiens* complex at all [6, 9]. Additionally, it is unclear how these two species are related, although early protein work suggested that they are more aligned with one another than to other members of the complex [15].

Another unresolved question in the *Cx. pipiens* complex is the evolutionary origins of the Asian endemic taxon, *pallens.* It has been postulated that the *pallens* form may be generated from ongoing hybridization between *pipiens* and *quinquefasciatus* in this region [16, 17]. However, there has been some question of this hypothesis due to the limited distribution of *pipiens* in East Asia [14, 18], although morphologically indistinguishable *molestus* is found throughout the region in urban areas (e.g. [19–23]). The hypothesis that *pallens* arose from hybridization between *quinquefasciatus* and *molestus* also presents a challenge however, as neither *quinquefasciatus* nor *molestus* can enter a diapause state, whereas *pallens* will diapause [24].

Studies of hybridization between *pallens*, *quinquefasciatus* and *molestus* in Southeast Asia indicate that mating between the three taxa can occur in the laboratory, but hybrids often lay fewer eggs and have reduced egg viability (e.g. [19, 20]). Correspondingly, families reared from naturally occurring hybrids between *pallens* and *molestus* in Japan were found to have lower fitness than families from either parental taxon [22]. Natural hybridization between *pallens* and *quinquefasciatus* has also been shown [25]. However, due to complex, asymmetrical patterns of genetic introgression the authors of this study concluded that *pallens* is unlikely to be a simple hybrid between the two taxa. An alternative hypothesis is that *pallens* derives from relatively older hybridization, after which it diverged as a distinct taxon, with likely occasional introgression from other taxa [25]. An assessment of possible hybrid origins, either recent or more ancient, is needed to elucidate the nature of the *pallens* taxon. If it is the result of relatively older hybridization events, the extent to which *pallens* has independently diverged is also unknown.

A third issue within the *Cx. pipiens* complex is the evolutionary origins and taxonomic status of *molestus*. Across most of its range, particularly in temperate regions, *molestus* is highly adapted to urban environments and correspondingly shows extensive ecological divergence to its presumed sister taxon, *pipiens* (reviewed in Vinogradova [14]). These divergent traits include an ability to lay eggs without a blood meal (autogeny), a willingness to mate in enclosed spaces (stenogamy), an absence of diapause, and variation in host preferences. However, it remains unclear whether *molestus* is simply an urban form of *pipiens* that can arise when *pipiens* adapts to cities, or conversely whether it has one or a few, distinct evolutionary origins.

Early behavioral and morphological observations suggested that *molestus* forms in North America likely originated locally and differed from European *molestus* [26]. In agreement with this hypothesis, recent analyses using microsatellites as well as restriction fragment length polymorphisms, concluded that North American *molestus* samples from New York City and Chicago were each more genetically similar to local *pipiens* populations than they were either to each other or to Old World *molestus* [27–29]. Additional work examining California populations of *Culex* also found evidence suggesting *molestus* populations in the USA are genetically distinct from *pipiens*, but also divergent from one another [30, 31].

However, contrasting work found that Old World *molestus* (Europe, Asia, Africa and Australia) were distinct from both European and North American *pipiens* [32]. This research also showed that *pipiens* from the USA were distinct from European *pipiens*, and observed that these *pipiens* have a unique genetic background which included both Old World *pipiens* and *molestus* ancestry. These results suggested either that the introduction of *pipiens* and *molestus* into North America were separate events, or that it was a hybrid form that was the original colonist. Additional microsatellite studies showed *molestus* specimens from Europe, the USA and Jordan are genetically more similar to one another than any is to *pipiens* [33, 34]. This result strongly suggests that these *molestus* share a common origin. Given contrasting findings regarding the origins of *molestus* mosquitoes, it presently remains unclear if *molestus* populations are globally monophyletic and genetically distinct from *pipiens*, or whether they are simply divergent ecological forms of *pipiens*.

Information that may address the above broad questions has practical importance and potential applications as mosquitoes in the *Cx. pipiens* complex are major vectors of several diseases that negatively impact humans such as West Nile virus and St. Louis encephalitis [35]. The degree to which complex members prefer to feed on birds, humans and/or other mammals varies [14, 35] and populations associated with distinguishable taxa also appear to vary in their competence as disease vectors [36, 37]. This variation in host preference and vector competence makes taxonomic designations and knowledge of genetic exchange important for understanding and potentially mitigating the transmission of diseases by these mosquitoes.

The aim of this work was to bring together the many existing next-generation sequencing datasets for the *Culex pipiens* complex to assess patterns of genetic diversity and divergence. The data available proved to have a near global distribution in sampling, allowing us to examine broad relationships among these taxa. We also aimed to address the specific questions posed above. Although limited in scope, our findings do provide support for many past taxonomic inferences in this complex. Critically, they also reveal several novel observations that warrant future investigation.

## Methods

### Data

The data used in this study predominately consisted of genomic and transcriptomic Illumina reads publicly available from the National Center for Biotechnology Information’s Short Read Archive database (NCBI-SRA; https://www.ncbi.nlm.nih.gov/sra). To locate these data, we first used a keyword search for ‘*Culex*’, and then limited potential datasets to only those stated to be from mosquitoes in the *Culex pipiens* complex with greater than 10 million reads and source population data, either as wild-collected samples or laboratory-maintained samples of known and limited geographical origin (Table 1, Additional file 1: Table S1). We also included data (as sequence traces) from the first publicly available *quinquefasciatus* genome assembly [49].

**Table 1:** Samples used in this study with taxon reported in the literature and the taxonomic designation determined here through our ADMIXTURE analyses

Sample code	Reported taxon	Ancestry assignment (K = 5)	Country	City	Reference
A_AUS_01	*australicus*	Australian endemic	Australia	Siesta Park	[38]
A_AUS_02	*australicus*	Australian endemic	Australia	South Guildford	[38]
A_AUS_03	*australicus*	Australian endemic	Australia	Point Douro	[38]
G_AUS_01	*globocoxitus*	Australian endemic	Australia	South Guildford	[38]
G_AUS_02	*globocoxitus*	Australian endemic	Australia	Siesta Park	[38]
G_AUS_03	*globocoxitus*	Australian endemic	Australia	Point Douro	[38]
L_CSA_01	*pallens*	Admixed	China	Zhu-Shang	[39]
L_CSA_02	*pallens*	Admixed	China	Nanjing	[40]
L_CSA_03	*pallens*	*pallens*	China	Dalian	[40]
L_CSA_04	*pallens*	*pallens*	China	Tongzhou (Beijing)	[40]
L_CSA_05	*pallens*	*pallens*	China	Chaoyang (Beijing)	[40]
L_CSA_06	*pallens*	*pallens*	China	Haidian (Beijing)	[40]
L_CSA_07	*pallens*	*pallens*	China	Shunyi (Beijing)	[40]
L_CSA_08	*pallens*	*pallens*	China	Shijingshan (Beijing)	[40]
M_EMD_01	*molestus*	Admixed	Germany*	Hamburg*	[41]
M_EMD_02	*molestus*	*pipiens-molestus* (EMD)	Russia	Aleksin	[42]
M_EMD_03	*molestus*	Admixed	Russia	Moscow	[42]
M_NCA_01	*molestus*	Admixed	USA (CA)	Sacremento	[42]
M_NCA_02	*molestus*	*pipiens-molestus* (NCA)	USA (NY)	New York	This study
M_NCA_03	*molestus*	*pipiens-molestus* (NCA)	USA (IL)	Chicago	This study
M_NCA_04	*molestus*	*pipiens-molestus* (NCA)	USA (NY)	New York	[43]
P_EMD_01	*pipiens*	*pipiens-molestus* (EMD)	Germany	Baden-Wurttemberg	[43]
P_EMD_02	*pipiens*	*pipiens-molestus* (EMD)	France	Ganges	[39]
P_EMD_03	*pipiens*	Admixed	France	Montpellier	[39]
P_EMD_04	*pipiens*	*pipiens-molestus* (EMD)	Algeria	Lac des Oiseaux	[39]
P_EMD_05	*pipiens*	Admixed	Israel	Tel Aviv	[39]
P_EMD_06	*pipiens*	*pipiens-molestus* (EMD)	Tunisia	Grombalia	[39]
P_EMD_07	*pipiens*	*pipiens-molestus* (EMD)	Tunisia	El Kef	[44]
P_EMD_08	*pipiens*	*pipiens-molestus* (EMD)	Russia	Aleksin	[42]
P_EMD_09	*pipiens*	*pipiens-molestus* (EMD)	Tunisia	Ayed	[44]
P_EMD_10	*pipiens*	*pipiens-molestus* (EMD)	Tunisia	Grombalia	[44]
P_EMD_11	*pipiens*	*pipiens-molestus* (EMD)	Tunisia	Jedaida	[44]
P_EMD_12	*pipiens*	*pipiens-molestus* (EMD)	Tunisia	Azib	[44]
P_EMD_13	*pipiens*	*pipiens-molestus (EMD)*	Tunisia	Utique	[44]
P_NCA_01	*pipiens*	*pipiens-molestus* (NCA)	USA (CA)	San Francisco	[45]
P_NCA_02	*pipiens*	Admixed	USA (OH)	Columbus	[46]
P_NCA_03	*pipiens*	Admixed	USA (NJ)	Montclair	This study
P_NCA_04	*pipiens*	*pipiens-molestus* (NCA)	USA (CA)	Bolinas	[45]
P_NCA_05	*pipiens*	*pipiens-molestus* (NCA)	USA (CA)	Stinson Beach	[45]
P_NCA_06	*pipiens*	*pipiens-molestus* (NCA)	USA (CA)	San Rafael	[45]
Q_AUS_01	*quinquefasciatus*	Admixed	Australia	South Guildford	[38]
Q_CSA_01	*quinquefasciatus*	*quinquefasciatus*	China	Haikou	[40]
Q_CSA_02	*quinquefasciatus*	*pallens*	China	Guangzhou	[40]
Q_CSA_03	*quinquefasciatus*	*quinquefasciatus*	Philippines	Manila	[39]
Q_CSA_04	*quinquefasciatus*	*quinquefasciatus*	China	Haikou	[40]
Q_CSA_05	*quinquefasciatus*	*quinquefasciatus*	China	Haikou	[40]
Q_CSA_06	*quinquefasciatus*	*quinquefasciatus*	China	Haikou	[40]
Q_NCA_01	*quinquefasciatus*	*quinquefasciatus*	Costa Rica	Puerto Viejo de Talamanca	[39]
Q_NCA_02	*quinquefasciatus*	Admixed	USA (CA)	Merced	[47]
Q_NCA_03	*quinquefasciatus*	Admixed	USA (AL)	Huntsville	[48]
Q_SSA_01	*quinquefasciatus*	*quinquefasciatus*	Burkina Faso	Ouagadougou	[39]
Q_SSA_02	*quinquefasciatus*	*quinquefasciatus*	Réunion	Saint-Benoît	[39]
Q_SSA_03	*quinquefasciatus*	*quinquefasciatus*	South Africa	Johannesburg	[49]

*Notes*: The country and city the sample originated from is also given. For samples designated as ‘Admixed’, no cluster is represented at greater than 75% when K = 5. Admixed proportions are given in Additional file 1: Table S2. (*sample origin given in personal communication from A. C. Honnen)

Although identification of the mosquito samples used to generate the data employed here was done by vector biology experts, we proceeded in our analyses on the assumption that taxonomic designations may be erroneous. The majority of these samples are pools of many individual mosquitoes, ranging from less than ten to several hundred. Concerns have been raised about the accuracy of categorizing genetic variation in such datasets (e.g. [50–52]). However, these concerns focus predominantly on the identification of rare alleles and estimates of allele frequencies utilizing read counts. Confident characterization of rare alleles is necessary for examining signatures of selection and demographic change, neither of which was a goal of this study.

Rather than using read counts in pooled samples to approximate allele frequencies, within each sample we characterized bi-allelic sites as homozygous for the reference state, homozygous for the alternative state, or heterozygous (segregating in the sample). In effect this established a ‘population genotype’ that we argue is comparable to individual genotypes in non-pooled samples. While this limited the analyses available to us, given the variation in the number of pooled mosquitoes and sequence depth among the samples, we felt this was the most analytically defensible approach for our data.

As a supplement to publicly available data, we also sequenced the genomes of three additional *Culex* samples. One of these was a single adult female from a laboratory strain of *molestus* that derived from New York City, USA [43]. The second was an adult female *pipiens*, reared from a larva collected in an oviposition trap placed in a wooded area on the campus of Montclair State University in Passaic County, New Jersey, USA. The nearest known natural population of *molestus* to this location is New York City, approximately 20 km away. We did not test whether this female was autogenic, or displayed any other traits which may have been indicative of *molestus* ancestry. DNA from both these samples was extracted using a standard phenol-chloroform protocol, then sequencing libraries were generated using the Nextera DNA Flex Library Prep Kit (Illumina, San Diego, USA). These libraries were sequenced on an Illumina HiSeq X Ten sequencer at the New York Genome Center (one lane per sample).

Our third dataset was generated from a single male *molestus* that was part of an inbred line (nine generations of sibling mating). The original population was collected in Calumet (Chicago), Illinois, USA [53]. Sequencing was performed at the North Carolina State University Genomic Sciences Laboratory on an Illumina HiSeq 2500 in Rapid Run mode. These data are available in the Short Read Archive database (BioProject: PRJNA561911).

### Read mapping and variant calling

Using the program Trim Galore (https://github.com/FelixKrueger/TrimGalore), we first trimmed the bases from read ends with quality scores (Q score) less than 20, then removed reads that were less than 30 bases long after trimming. For paired read datasets, after trimming all unpaired reads were also removed. Quality trimming was done for all samples that consisted of Illumina reads (all but the South African *quinquefasciatus* sample).

For samples that derived from messenger RNA (i.e. RNA-seq data), we mapped the trimmed reads to a high-quality reference genome of *quinquefasciatus* (GSE95797_CpipJ3 [54]), using the program Star v. 2.5.2 with 2 pass mapping [55, 56]. For this, the reads were first mapped to the genome with default program parameters. Next, all splice junctions that were detected in the first pass were merged using a splice junction database overhang value of 75 (–sjdbOverhang 75). In the same step we removed likely false positives and generated an updated reference genome index. Lastly, we remapped the reads using this new genome index. For genomic datasets (including the South African *quinquefasciatus* sample) we mapped reads to the same reference genome as for RNA-seq data (see above), using the program BWA-MEM v. 0.7.15 with default settings [57].

For samples of both data types, after mapping we identified and marked read duplicates using the tool MarkDuplicates from Picard v. 1.77 (http://broadinstitute.github.io/picard/). This was followed by indel realignment using IndelRealigner from the Genome Analysis Toolkit (‘GATK’) v. 3.8 [58]. Independently for each sample, we called variant sites using GATK’s HaplotypeCaller (specific flags: –emitRefConfidence GVCF, –variant_index_type LINEAR, –variant_index_parameter 128000 -rf BadCigar). For pooled samples, ploidy was set to the number of individuals that made up that sample. When a range was reported, the highest value given was used. The resulting gVCFs (one per sample) were then combined and the samples collectively genotyped using GATK’s GenotypeGVCFs function.

We retained only bi-allelic, single nucleotide polymorphisms (SNPs) located on one of the three *Culex* chromosomes and present in all samples with a read depth of at least five reads per sample. Because our focus was exclusively on population and taxon relationships, we wanted to utilize genetic variants that were effectively ‘neutral’ (i.e. have not experienced direct, divergent selection between taxa). Therefore, we generated a primary dataset that consisted of only four-fold degenerate (synonymous) sites. These were the best available neutral variant type available from this dataset, even though such sites may not be completely neutral due to codon usage bias [59] as well as other types of direct or indirect selection [60, 61].

To locate four-fold degenerate sites, we first produced an annotation of the *quinquefasciatus* reference genome using the program BRAKER2 [62] and the protein predictions from the first publicly available *quinquefasciatus* genome assembly and annotation [49]. We then used the program SnpEff v. 4.3 [63] to identify silent (synonymous) segregating variants. Finally, we used BCFtools v. 1.9 [64] to filter out all sites except those that were four-fold degenerative. We considered this to be our primary dataset although we also performed all analyses using our more extensive, second dataset that contained all bi-allelic, segregating variants.

For both datasets, we removed SNPs that had a quality by depth less than 2 (QD < 2.0), Fisher strand bias greater than 40 (FS > 40.0), mapping quality less than 55 (MQ < 55.0), mapping quality rank sum less than − 0.2 (MQRankSum < − 0.2), read position rank sum less than − 2 (ReadPosRankSum < − 2.0), and a strand odds ratio greater than 3 (SOR > 3.0). All filtering options were based on the developer’s recommended cut-offs, with more stringent adjustments for FS, MQ, MQRankSum and ReadPosRankSum based on the observed distributions for these parameters (Additional file 2: Figure S1). We next used VCFtools v. 0.1.17 [65] to remove SNPs that were not in Hardy-Weinberg equilibrium using a *P*-value of 10^−4^. We also removed any SNP with a minor allele frequency less than 5%. Finally, as linkage between SNPs could impact observations of population structure and connectivity [66], we used the program PLINK v. 1.90b6.6 [67] to remove SNPs with a pairwise squared correlation (r^2^) greater than 50% within sliding windows of 50 SNPs at 10 SNP increments between windows [68].

### Admixture and population structure

Because mosquitoes within the *Culex pipiens* species complex are notoriously challenging to accurately identify to taxon, our initial analyses avoided the use of any *a priori* taxonomic designations of the samples. Rather, we focused on genetic comparisons that did not require sample taxon labels.

First, we used a principal component analysis (PCA) to investigate genetic clustering among all samples. We also examined clustering after excluding the samples designated as either of the two Australian endemic taxa (*australicus* or *globocoxitus*). These PCAs were carried out using the program PLINK v. 1.90b6.6 [67], and the results were visualized using R v. 3.5.1 [69], with sample coding based on the published taxonomic designations.

Next, we evaluated genetic structure and patterns of genetic exchange with a maximum likelihood approach using the program ADMIXTURE v. 1.3.0 [70], examining potential clusters (K) from one to seven. Each K value was run 20 independent times with different starting seed values used for each run. Across K values, means observed for the standard error of the 5-fold cross-validation error estimate were compared to identify the number of taxa best supported by our data. Generally, smaller values suggest more strongly supported clusters [71]. We used the online version of CLUMPAK [72] with default settings to determine the average q-matrix cluster assignment for each sample, at each K value.

To complement our ADMIXTURE analyses, we used the program STRUCTURE v. 2.3.4 [66] to examine population clustering among our samples in a Bayesian framework. Many studies have shown that uneven sampling among possibly structured populations may bias STRUCTURE results (e.g. [73–75]). In our dataset, we had substantial variation in taxonomic and geographical representation. However, given the complex nature of our dataset, it was unclear how best to resolve the issue of uneven sampling among populations and taxa. Therefore, we took a straightforward approach and removed all but one representative of geographically proximate samples of the same reported taxonomic designation (see Additional file 1: Table S1). Geographical proximity was defined as two locations being within 100 km of each other. When two or more samples fit this definition, the sample with the lowest percentage of missing variants in our unfiltered dataset was retained (data not shown). We assessed the proportion of missing variants per sample using VCFtools v. 0.1.17 [65]. After this sample reduction, 35 samples remained for our STRUCTURE analysis.

With this reduced number of samples, we examined the potential number of clusters (K) represented in our datasets from one to seven, using the admixture model and applying a ‛burn-in’ period of 10,000 followed by 50,000 replicates. Each value of K was run five independent times. The program STRUCTURE HARVESTER v. 0.6.94 [76] was used to analyze these results and apply Evanno’s DK [77] to estimate the number of clusters best supported by our data. We also examined the support for each K using median posterior probabilities across replicates, followed by an application of Bayes’ rule [78]. This was done using the online version of CLUMPAK [72] with default settings. CLUMPAK was also used to determine the average q-matrix cluster assignment for each sample, at each value of K.

### Phylogenetic analysis

We used a maximum likelihood (ML) approach to examine phylogenetic relationships among our samples. Our analysis with four-fold degenerate sites used a transversional model of mutation with a proportion of invariable sites and a gamma distribution of rate heterogeneity (TVM + I + Γ [79]). We applied a generalized time reversible model with a gamma distribution of rate heterogeneity (GTR + Γ [80]) to our dataset containing all segregating sites. The evolutionary models for both datasets were determined to be the best-fit to the data based on AIC score using jModelTest v. 2.1.10 [81, 82]. Our ML analysis for the four-fold degenerative site dataset was carried out with PhyML v. 3.1 [83], with 100 non-parametric bootstrap replicates to determine confidence values for the observed clades. Because of a greater amount of data, our ML analysis for the dataset containing all segregating sites was run in RAxML v. 8.2.12 [84], again with 100 non-parametric bootstrap replicates to determine confidence values.

### Taxa differentiation

Our ADMIXTURE and STRUCTURE analyses suggested that the samples in our datasets may represent five distinct genetic clusters (with the possibility for admixture between them; see Results). These clusters correlate with an Australian-endemic, *quinquefasciatus, pallens* and two *pipiens* clusters. The *pipiens* clusters correspond to North American and Europe/Mediterranean populations respectively. Among these clusters there is substantial admixture, but each cluster had multiple (≥ 6) samples with 100% cluster membership (Table 1, Additional file 1: Tables S2, S3). Using these 100% membership samples, we examined taxonomic differentiation by calculating the fixation index (F_st_) between the samples in these five taxonomic clusters. We also calculated F_st_ using the samples reported to be from each of the two Australian-endemic taxa.

There have been several approaches developed to calculate the fixation index (F_st_) between populations using data from pooled individuals (e.g. [85–87]). Broadly these are designed for use only with pooled genomic DNA, with an assumption of equivalent amounts of DNA per individual per pool, and similar numbers of individuals per pool (e.g. [85] but see [87]). The samples used here included both individual and pooled sequencing efforts, as well as large variation in the number of individuals within each pooled sample (Additional file 1: Table S1). Hivert et al. [87] showed a high degree of correlation between their explicit estimates of F_st_ using pooled-sequencing data and similar estimates using the method of Weir & Cockerham [88] for multilocus data from single samples. Additionally, we did not use single pools of a population sample to estimate F_st_, but rather multiple pools of individuals for each taxon of interest. For these reasons, we calculated pairwise F_st_ between each of the five sample clusters with the method of Weir & Cockerham [88], using VCFtools v. 0.1.17 [65]. We report both the unweighted and weighted estimates. Unweighted estimates should be less biased by unequal samples sizes, whereas weighted estimates are less affected by rare variants [89].

## Results

### Data

After filtering, our four-fold degenerative sites dataset retained 6282 unlinked, single nucleotide, bi-allelic variants. Our dataset with all segregating sites retained 16,105 unlinked, single nucleotide, bi-allelic variants after filtering. These SNPs were generally well distributed across the three *Culex* chromosomes, with only substantial reductions in representation around the centromeres (Additional file 2: Figure S2).

### Admixture and population structure

**Fig. 1:**
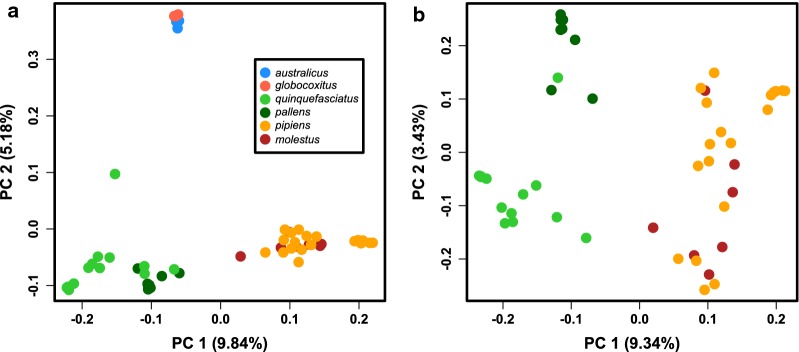
Principal components analysis (PCA) using four-fold degenerate sites with reported samples from all six described members of the *Culex pipiens* complex (**a**) and with a four-taxon set that excluded the reported Australian endemic taxa, *australicus* and *globocoxitus* (**b**). These PCAs were implemented with PLINK and plotted in R. Shown are the first two PCs. Colors corresponding to the different reported taxa are consistent between the two PCAs

In our PCA using all samples and the dataset of four-fold degenerate sites, samples with the published taxonomic designation of *pipiens* or *molestus* formed a cluster distinct from the other samples along PC 1 (Fig. 1a). Along PC 2 the samples with a taxonomic designation of either *australicus* or *globocoxitus* (i.e. the Australian endemic taxa), separated from samples designated as *quinquefasciatus* and *pallens*, with the one Australian sample reported as *quinquefasciatus* being intermediate between these two clusters. When we looked at just the samples excluding those reported to be from an Australian endemic taxon, we again observed that samples designated as *quinquefasciatus*/*pallens* were distinct from those designated as *pipiens/molestus* along PC 1 (Fig. 1b). However, we also detected a degree of separation between *quinquefasciatus* and *pallens* along PC 2. One sample reported as *quinquefasciatus* (from China) was grouped within this distinct *pallens* cluster. Nearly identical patterns were observed in our principal component analyses utilizing the ‘all segregating sites’ dataset (Additional file 2: Figure S3).

**Fig. 2:**
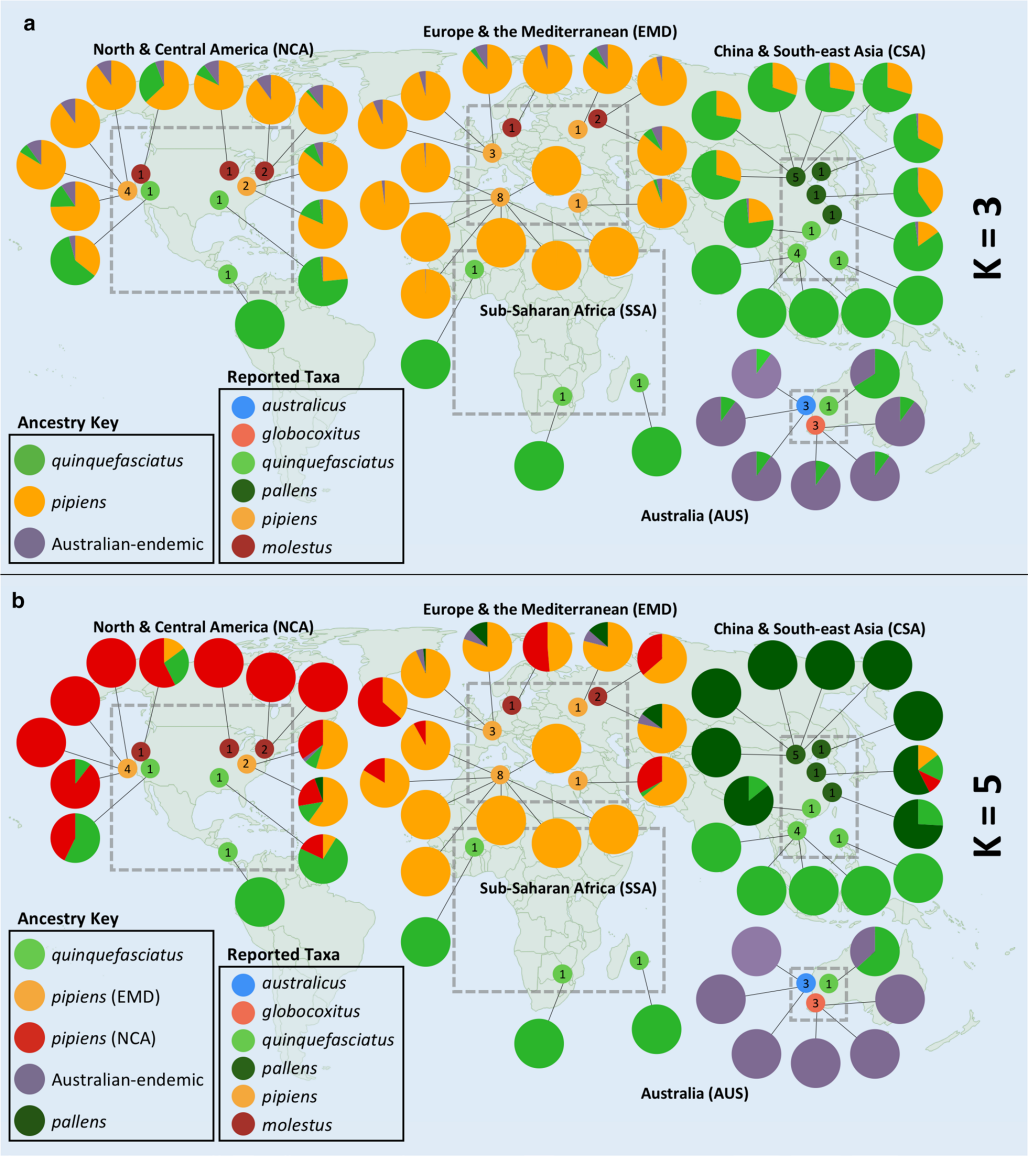
World maps showing the described collection locations of samples (small circles inside gray boxes) and the relative proportions of three (**a**) or five (**b**) inferred populations as determined in our ADMIXTURE analysis (large circles), using four-fold degenerate sites. Each sample’s taxonomic designation was based on that reported in the literature (see Table 1, Additional file 1: Table S1). For the ADMIXTURE results the proportion of each color in the circle corresponds to the amount of cluster-associated ancestry. Note that for our sample designations, we defined five broad geographical regions, indicated on the map by the dashed gray boxes

In our ADMIXTURE analysis, the lowest mean cross-validation (CV) error values for both datasets occurred when K = 3 (Additional file 1: Table S4, Additional file 2: Figure S4). These three groups broadly correspond to an Australian cluster that includes samples designated as *australicus* and *globocoxitus*, a *quinquefasciatus* cluster, and a *pipiens* cluster that includes samples designated as *molestus* (Fig. 2a, Additional file 2: Figures S5, S6). In both datasets, most of the samples reported as *pallens* have a predominately *quinquefasciatus-*like genetic background, but contain 15.3% to 40.0% genetic background corresponding to the *pipiens* cluster (average: 29.0%, these and proceeding values from the ‘four-fold degenerate sites’ dataset). We also observed that the one Australian sample reported as *quinquefasciatus* had a substantial proportion of Australian-endemic ancestry (34.0%) suggesting possible genetic exchange with either *australicus* or *globocoxitus*. It was not possible to differentiate between *australicus* and *globocoxitus* ancestry in these analyses. Our two samples reported as *quinquefasciatus* from North America had 23.4% (California) and 35.7% (Alabama) *pipiens*-like background, and the reported *molestus* sample from California had a predominately *pipiens*-like background but additionally had 31% *quinquefasciatus*-like ancestry. Broadly, nearly all *Culex* samples from North America showed greater levels of population admixture than those from Europe, the Mediterranean and sub-Saharan Africa.

For K = 4, we observed subdivision in the *pipiens*/*molestus* cluster that roughly divided the North American samples from those of Europe and the Mediterranean (Additional file 2: Figures S5, S6). However, we found evidence of both New World and Old World ancestry in the two eastern North American *pipiens* samples, the one California *molestus* sample, two of the three European samples designated as *molestus*, and five of the 13 European and Mediterranean samples designated as *pipiens*.

The samples reported to be *pallens* revealed a unique genetic signature at K = 5, with most samples exhibiting 100% *pallens*-like ancestry (Fig. 2b, Additional file 2: Figures S5, S6). The two reported *pallens* samples from more southernly parts of China harbored some *quinquefasciatus*-like ancestry, and one of these also had genetic variation that corresponds to both a European/Mediterranean and North American *pipiens*-like genetic background. The most northerly sample from China reported as *quinquefasciatus* had a predominately *pallens*-like background (85.8%), with the remaining genetic variation coming from *quinquefasciatus*. This suggests the individual mosquitoes that made up this pooled sample may have been mischaracterized. At K = 6, the *pipiens* and *molestus* samples were further subdivided, and with K = 7 the reported North American *molestus* samples exhibited a unique genetic signature. Samples that had less than 75% genetic ancestry from any of the five clusters at K = 5 are classified as ‘Admixed’ in Table 1 and Additional file 1: Table S1. The specific ancestry proportions are given in Additional file 1: Table S2 for the ‘four-fold degenerate sites’ dataset and in Additional file 1: Table S3 in the ‘all segregating sites’ dataset.

**Fig. 3:**
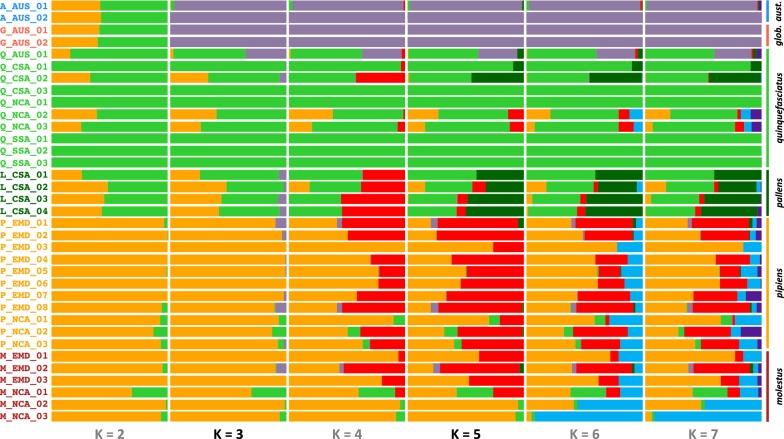
STRUCTURE bar plots for the samples in our subsampled dataset plotted for genetic clusters (K) from two through seven, using four-fold degenerate sites. Each horizontal bar represents one sample. The relative proportions of each color indicate the proportion of genetic diversity assigned to that cluster. Sample designations are reported along the left y-axis. Taxon groups are reported along the right y-axis. The two best-supported K values are given in black text at the bottom (K = 3 for Evanno’s DK; K = 5 for median posterior probability). For additional sample details, see Additional file 1: Table S1

For the STRUCTURE results, three clusters were best supported in both datasets (Additional file 1: Table S5) when we applied Evanno’s DK [77]. This agreed with our ADMIXTURE analyses. These three groups again corresponded to an Australian-endemic cluster, a *quinquefasciatus* cluster and a *pipiens*/*molestus* cluster (Fig. 3, Additional file 2: Figure S7). The reported *pallens* samples had 47–68% *quinquefasciatus*-like association and 25–48% *pipiens*-like association when the data were divided between three clusters (values from our ‘four-fold degenerate sites’ analysis). At K = 4, portions of the reported *molestus*, *pipiens* and *pallens* samples became distinct, although there were no clear geographical or taxonomic associations. In contrast to Evanno’s DK, the median posterior probability of each K value across replicates suggested that K = 5 was the best supported number of clusters (Additional file 1: Table S6). This corresponds to an Australian-endemic cluster, a *quinquefasciatus* cluster, a *pallens* cluster and two distinct clusters among the *pipiens* samples, again with no clear taxonomic or geographical association (although the two reported eastern North American *molestus* samples exhibited some distinctiveness). At higher values of K, smaller proportions of the samples were distinguished with no clear taxonomic or geographical patterns emerging (Fig. 3, Additional file 2: Figure S7).

### Phylogenetic analysis

**Fig. 4:**
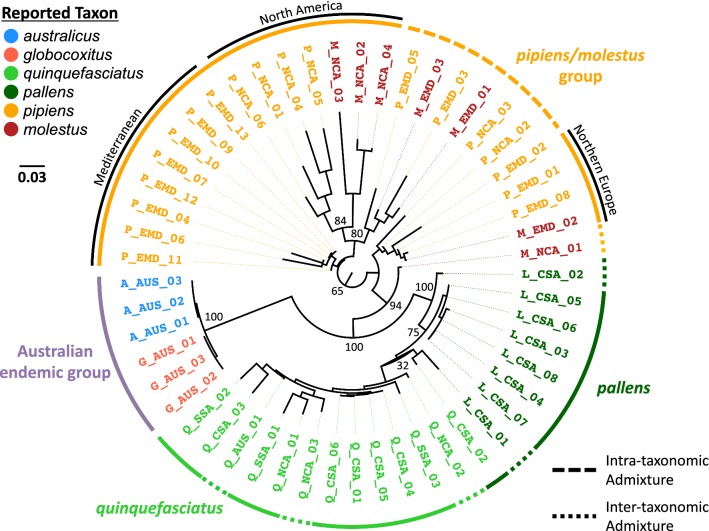
Maximum likelihood phylogeny using four-fold degenerate sites and a transversional mutation model with a proportion of invariable sites and a gamma distribution of rate heterogeneity (TVM + I + Γ; [79]). The colors for the branch tip labels correspond to the six different taxa in this study. The numbers at the major branch nodes indicate bootstrap support for each bifurcation in the tree (out of 100). The three-letter code in the middle of each sample name indicates its geographical region of origin (see Additional file 1: Table S1 for additional sample details). Samples under a broad dashed line were determined to be intra-taxonomically admixed (*pipiens* and *molestus* only). Samples under a fine dashed line were determined to be inter-taxonomically admixed. Within the *pipiens* and *molestus* samples, three broad geographical clusters are defined: North America, Mediterranean and northern Europe (including Russia)

Our maximum-likelihood phylogenetic analyses broadly correlated with our analyses of taxa differentiation and clustering with both datasets (Fig. 4, Additional file 2: Figure S8). In particular, we saw two broad clusters, one containing the reported *globocoxitus*, *australicus*, *quinquefasciatus* and *pallens* samples, and a second containing the reported *pipiens* and *molestus* samples. The *pipiens* and *molestus* samples split into three rough geographical groups, rather than by taxon. These approximately correlate with a North American cluster, a Mediterranean cluster, and a northern European (including Russia) cluster. However, as indicated by our ADMIXTURE and STRUCTURE analyses, throughout the *pipiens*/*molestus* clade there is extensive intra-taxonomic genetic exchange and admixture.

In contrast to the *pipiens*/*molestus* branches, all but one designated *quinquefasciatus* sample formed a distinct, monophyletic cluster, as did the Australian endemic taxa. The bifurcation between the Australian endemic taxa and *quinquefasciatus*/*pallens* was strongly supported (100/100). Within the Australian-endemic/*quinquefasciatus*/*pallens* branch of the tree, the Australian endemics were distinct from *quinquefasciatus* and *pallens* with complete bootstrap support (100/100 trees). The reported *quinquefasciatus* samples mostly formed a monophyletic clade distinct from the *pallens* samples (one designated *quinquefasciatus* from China clustered with the *pallens*).

### Taxa differentiation

In all pairwise comparisons across both datasets, our estimates of unweighted F_st_ values were less than the weighted estimates (Table 2, Additional file 1: Table S7). Values were similar between estimates calculated using only four-fold degenerate sites and those found using all segregating sites (maximum difference between datasets: ± 0.010). Therefore, we will here only report F_st_ estimates from our ‘four-fold degenerate sites’ dataset. Unweighted F_st_ values ranged from 0.116 to 0.298, with the average being 0.226 (SD: 0.057). Weighted F_st_ values ranged from 0.137 to 0.460, with the average being 0.322 (SD: 0.106). The lowest F_st_ values for both the weighted and unweighted estimates were between *pipiens* samples with a North American (NCA) ancestry and those with a European/Mediterranean (EMD) ancestry (unweighted: 0.116; weighted: 0.136). The highest F_st_ values among our unweighted estimates were between *quinquefasciatus* and the *pipiens* samples with a European/Mediterranean ancestry (0.298). Among our weighted estimates, the highest F_st_ values were between *quinquefasciatus* and the Australian endemic taxa (0.470). Between the two Australian-endemic taxa the unweighted F_st_ estimate was 0.056 and the weighted estimate was 0.078.

**Table 2:** Pairwise unweighted and weighted F_st_ values [88] for each taxonomic cluster as determined by the ADMIXTURE analysis, using our four-fold degenerate site dataset and samples with 100% cluster assignment (see Additional file 1: Tables S1, S2)

	Australian endemics	*quinquefasciatus*	*pallens*	*pipiens* (NCA)	*pipiens* (EMD)
Australian endemics	–	0.280	0.250	0.241	0.267
*quinquefasciatus*	0.470	–	0.182	0.252	0.298
*pallens*	0.366	0.223	–	0.178	0.191
*pipiens* (NCA)	0.410	0.399	0.251	–	0.116
*pipiens* (EMD)	0.355	0.384	0.228	0.136	–

*Note*: Unweighted values appear above the diagonal and weighted values appear below the diagonal

*Abbreviations*: NCA, North American Cluster; EMD, Europe and the Mediterranean Cluster

## Discussion

Despite the assortment of sampling and sequencing strategies used to generate the data utilized here, this study revealed broad taxonomic relationships within the *Culex* species complex. It is evident that these taxa have not diverged substantially at the genomic level, but rather maintain a cohesiveness, likely facilitated by extensive genetic exchange. Considering these observations, it is not surprising that this complex has continued to elude clear answers regarding taxonomic relationships among its members. Nevertheless, this study convincingly shows some consistent associations and relationships among these *Culex* mosquitoes that provide a better understanding of the complex overall.

### What is the relationship of the Australian endemic taxa to the rest of the *Cx. pipiens* complex?

Although the two Australian endemic taxa, *australicus* and *globocoxitus*, have generally been placed within the *Culex pipiens* complex, there has been discussion as to whether they are true members or rather whether one or both is a sister group [6, 8, 9]. Furthermore, their evolutionary origins have remained obscure, as has their relationship to one another [7, 15]. We observed in our principal component analyses a clear degree of cluster separation between the Australian endemic taxa and the other members of the group along the second principal component axis. Additionally, F_st_ values were highest between the Australian taxa and the other four genetic clusters.

These observations suggest that within the complex, *australicus* and *globocoxitus* are genetically distinct, and lend support to a relatively early separation [13, 14]. However, within our phylogenetic analyses, the Australian clade of samples does not fall outside of the remaining samples (i.e. is sister to them), but rather branches from the *quinquefasciatus* clade, after its split from the *pipiens* clades. This observation suggests that the Australian endemic taxa may have diverged from *quinquefasciatus* in Australia, after the separation between *quinquefasciatus* and *pipiens* as has been previously proposed [13]. If this scenario is correct, it means that these two Australian mosquitoes belong firmly within the *Cx. pipiens* complex. A second relevant observation is that *australicus* and *globocoxitus* appear to be sister taxa, and furthermore to have diverged relatively recently. The F_st_ values for the samples reported from each of these two taxa were 0.056 (unweighted) and 0.078 (weighted); values that are lower than those observed for the analyses of genetic divergence between the five distinct genetic clusters. These observations support earlier findings of a close kinship between these two species from protein data [15]. We have made no attempt to estimate divergence times here given the complexities of our dataset. However, the relatively short branch lengths in our phylogeny as well as the low F_st_ values, suggest that the two Australian taxa shared a common ancestor that is likely more recent than those of the other members of the complex, with the possible exception of *pipiens* and *molestus*. It is also possible that extensive genetic exchange between *australicus* and *globocoxitus* has acted to reduce genetic differentiation between them. Despite either recent divergence and/or ongoing genetic exchange, we see clear evidence that they are distinct from one another in our admixture and phylogenetic analyses, supporting known differences in ecology, morphology and behavior [10–13].

Yet further evidence that *australicus* and *globocoxitus* belong within the *Cx. pipiens* complex comes from the Australian *quinquefasciatus* sample in this study. This sample (which was a pool of 5–10 individual mosquitoes) appears to show evidence of introgression from one of the two Australian endemic taxa, suggesting that these taxa naturally hybridize (Figs. 1, 2, 3, Additional file 2: Figures S3, S5–S7). This is further evidence that the Australian endemic taxa are closely aligned with *quinquefasciatus*. However, an alternative explanation is that the pool of mosquitoes that comprised this sample contained one or more *australicus* or *globocoxitus* samples. These seems less likely though, as the samples were identified as *quinquefasciatus* using both morphological and molecular methods [38], and none of the pooled samples designated as *australicus* or *globocoxitus* from this same study show a similar signature of taxonomic admixture.

### Is *Cx. pipiens pallens* of hybrid origin?

In all analyses, the *pallens* samples consistently clustered most closely with those of *quinquefasciatus*. However, a comparison of F_st_ values between the *pallens*-, *quinquefasciatus*- and *pipiens-*clusters, suggests an interesting pattern. Specifically, unweighted and weighted F_st_ values between the *quinquefasciatus*- and the two *pipiens-*clusters (EMD/NCA) were 0.298/0.252 and 0.384/0.399, respectively (values from the ‘four-fold degenerate sites’ dataset). By contrast, between *pallens* and the two *pipiens-*clusters (EMD/NCA), values were 0.191/0.178 and 0.228/0.251 for unweighted and weighted F_st_. A lower degree of genetic divergence between *pallens* and *pipiens* (or *molestus* which was generally grouped within the *pipiens* clusters) may suggest recent genetic exchange between these taxa. Hybridization between *pallens* and *molestus* has been reported previously [22]. However, a non-mutually exclusive possibility is that *pallens* arose from hybridization between *quinquefasciatus* and *pipiens/molestus* at some point in the past and then subsequently diverged as a distinct taxonomic entity. Further support for this hypothesis comes from our clustering analyses. In our PCAs, the *pallens* samples did not fall intermediately between the *quinquefasciatus* and *pipiens/molestus* samples as might be expected if they were recent hybrids. Rather, they formed a relatively tight and distinct cluster. This is especially evident in the PCAs excluding the Australian endemic taxa (Fig. 1b, Additional file 2: Figure S3b).

In the ADMIXTURE analysis for K = 3 we observed that in all *pallens* samples most of the genetic background comes from *quinquefasciatus*, but a substantial proportion (25–48%) is aligned with a *pipiens/molestus* background (Fig. 2a). Most samples had slightly more than a quarter *pipiens/molestus* genetic background. Again, this consistency between samples suggests *pallens* is of relatively older hybrid origin, rather than a swarm of recent hybrids. Recent hybrids would likely have greater variance in the relative proportions of *quinquefasciatus* and *pipiens/molestus* genetic background [90]. When we look at larger K values, in particular five and above, we see that *pallens* becomes its own unique genetic cluster (Fig. 2b, Additional file 2: Figures S5, S6). This is further evidence that contemporarily, *pallens* is distinct and not a hybrid swarm. Both the mixture of *pipiens/molestus* and *quinquefasciatus* backgrounds at lower K values (three and four), and genetic distinctiveness at higher K values (five and above) is also seen in our STRUCTURE analysis (Fig. 3, Additional file 2: Figure S7). Lastly, in our phylogenetic analysis *quinquefasciatus* and *pallens* form mostly discrete clades.

Despite our results, the hypothesis that *pallens* formed through past hybridization between *quinquefasciatus* and either *pipiens* or *molestus* has clear biological challenges, depending on which was the second hybridizing taxon. First, as there are no known contemporary populations of *pipiens* in East Asia, it is presently unclear where hybridization between *quinquefasciatus* and *pipiens* could have occurred to form *pallens.* Conversely, if hybridization between *quinquefasciatus* and *molestus* produced the *pallens* form, the question arises of how an ability to enter diapause developed in *pallens* as neither *quinquefasciatus* nor *molestus* has an ability to diapause. Further support for an ‘ancient’ hybrid origin of *pallens* will require additional future analyses.

### Is *Cx. pipiens* f. *molestus* a distinct, monophyletic taxonomic entity?

Neither the reported *molestus* nor *pipiens* samples formed a monophyletic cluster in any analysis. However, more regionally we do see differences between the two taxa. In particular, the eastern North American samples of *molestus* appear distinct at K = 7 in our ADMIXTURE analyses and starting at K = 6 in our STRUCTURE analyses (Additional file 2: Figures S5-S7). Perhaps surprisingly, these reported *molestus* samples are most closely aligned with the reported western North American samples of *pipiens*. This may suggest that North American *molestus* arose first on the west coast of North America. This possibility is particularly intriguing given the complex genetics of *Cx. pipiens* taxa in California [30, 31, 34, 91], and the high prevalence of autogeny (ability to lay eggs without a blood meal) observed in central Californian *Culex* [30, 31, 34].

Our phylogenetic analyses also support a relatively close relationship between western North American *pipiens* and our North American *molestus* samples from Chicago and New York City. These eastern USA *molestus* samples formed a well-supported, distinct clade separate from the reported European *pipiens* and *molestus* samples, as well as the eastern North American *pipiens* (Fig. 4, Additional file 2: Figure S8). This result contrasts with the findings of Kothera et al. [28], who suggested that North American *molestus* samples from New York City and Chicago derived from local *pipiens* in each city. Interestingly, the sample designated as *molestus* from California is the most distinct among the reported *pipiens*/*molestus* samples. This is explained by the presence of substantial genetic ancestry from *quinquefasciatus* (Figs. 2, 3, Additional file 2: Figures S5, S7). Extensive hybridization between autogenous forms of *Culex* in California and *quinquefasciatus* has been previously observed [30, 31, 34].

The reported European *molestus* samples showed less distinctiveness in our ADMIXTURE and STRUCTURE analyses, but are broadly most closely related to one another in our phylogenetic analyses, with one reported *pipiens* sample from France falling within this clade and one sample from Russia placed distantly on the tree (Fig. 4, Additional file 2: Figure S8). We also found the single *pipiens* sample from Israel to be closely aligned with these samples. Interestingly, the four samples (two *molestus* and two *pipiens*) had high proportions of genetic ancestry most closely aligned with North American *pipiens* and *molestus*, and were the sister clade to our west coast *pipiens* and east coast *molestus* samples. It is notoriously difficult to distinguish *molestus* from *pipiens* morphologically, and accordingly it is possible the two *pipiens* samples in this cluster were mis-identified in the original studies. In addition to their presence in North America and Europe, *molestus* also occurs extensively in the Middle East [92].

Overall, our comparisons of New World and Old World *pipiens* and *molestus* broadly support the findings of Fonseca et al. [31], who showed that *pipiens* and *molestus* were genetically distinct. However, it also points toward the possibility of independent evolutionary origins for New World and Old World *molestus*, with additional influences of genetic exchange between *molestus* and *pipiens*. This result is surprising given that previously *molestus* specimens from Europe, the USA and Jordan were found to be most genetically similar to one another [33, 34], suggesting that globally, *molestus* may share a common origin. While the data examined here support multiple origins for *molestus*, our observations of extensive genetic exchange among all the taxa suggest this is best considered a tentative hypothesis. Many more samples will be needed to confidently resolve this question, with western North American *Culex* being of particular interest.

### Limitations of this study

Our reliance on predominately publicly available data meant this study necessarily had some limitations. Foremost, the sampling of taxa and populations was uneven with many locations missing that should be included in a more dedicated and robust study of the global *Cx. pipiens* complex. We also utilized a wide variety of data types, potentially bringing into question the reliability of our genetic variant calling. However, we feel this is not a true limitation of this study, as our rigorous variant filtering ensured that the datasets we utilized accurately captured patterns of diversity and divergence among these taxa. On the contrary, this study shows the utility of using publicly available data to answer questions of species relationships and evolutionary histories.

Further considering our use of publicly available data, the accuracy of taxonomic designations is of some concern. Individual mosquitoes within the *Cx. pipiens* complex are difficult to confidently assign to a specific taxon, especially *pipiens* and *molestus* which have no clear or consistent morphological differences [14]. Our use of many datasets that were of pooled samples may actually have negated some of this problem if the majority of the mosquitoes that went into each pool were of the designated taxon. Perhaps surprisingly, we see very little incongruence between taxonomic designations and sample clustering in our analyses. The one clear exception is a *quinquefasciatus* sample from China that appears to be *pallens*. Among our *pipiens* and *molestus* samples, it is impossible to determine if many of the taxonomic designations are incorrect within the context of this study. Nonetheless, all eastern USA *molestus* samples were determined to be autogenic [43, 53], as was the sample from Germany [41]. The *molestus* from the western USA and Russia were taxonomically assessed using molecular methods [42]. However, many of the *pipiens* samples were not confirmed using molecular methods nor assayed for possible *molestus*-like traits. Incorrectly identified taxonomic designations among the *pipiens* and *molestus* samples may at least partially explain the complex relationships, patterns of divergence, and signatures of admixture uncovered in this study.

It is also possible that the pooling of individual mosquitoes in many of our samples elevated observed rates of admixture. Certainly, if some or many of these pools contained multiple taxa, this would lead to an appearance that these samples were highly admixed. However, multiple observations suggest this alone does not explain the entirety of the observed genetic patterns here. First, in the most consistently admixed group, *pallens*, the samples were all comprised of pooled samples. Despite this, the proportionate contributions from a *quinquefasciatus* and *pipiens*/*molestus* genetic background remain remarkably consistent across broad geographical distances. This is strongly suggestive that the data are capturing intra-individual admixture patterns, not simply a mixture of taxonomic backgrounds at the population level. Secondly, several of our single-mosquito samples exhibited a high degree of admixture (e.g. New Jersey *pipiens*), indicating that substantial admixture occurs within individual mosquitoes. Finally, and perhaps most fundamentally, the sample pools were all produced by vector biology experts with substantial experience working with *Culex* mosquitoes (see Table 1, Additional file 1: Table S1 for references).

Lastly, there is the question of whether the molecular markers we utilized are ‘neutral’ (i.e. not under strong selective forces). Most of the analyses we performed assume that there is not strong selection acting on the segregating variants utilized. This was the motivation behind our generation of the ‘four-fold degenerate sites’ dataset. However, four-fold degenerate sites may still diverge between taxa due to differences in codon usage and/or selection at linked sites [59–61]. More broadly the segregating variants in our ‘all segregating sites’ dataset likely fall within exons or transcribed, untranslated regions (UTRs). As the taxa examined here are found in very different environments (e.g. tropical *vs* temperate), it is possible that a substantial proportion of these variants have diverged due to direct selection pressures or else selection on closely linked sites (in addition to the aforementioned codon bias). Such selection pressures could influence the distribution of alleles used in this study. However, these factors would likely work to increase levels of observed divergence between taxa and population substructure within broadly distributed taxa. Likewise, changes in allele frequencies in relation to demographic changes may also be a factor that could have influenced the patterns of divergence and admixture we described here, but again these would most likely act to increase divergence [93].

## Conclusions

As the amount of next-generation sequence data continues to increase, opportunities to combine discrete datasets to address important biological questions will grow. We used data from twelve different studies, combined with our own sequencing efforts, to carry out a global analysis of taxon relationships within the *Cx. pipiens* complex. Our results suggest that Australian endemic species share a unique evolutionary history. We also found evidence that *pallens* results from ancestral hybridization between *quinquefasciatus* and *pipiens*, and that it is presently a distinct evolutionary entity. This hypothesis warrants further examination. Finally, our results reveal that *molestus* may have had two distinct evolutionary origins, one in North America and one in Europe. We hope that these results, as well as the broad patterns of relationship uncovered in this study, will spur additional research into these areas. We also hope that the better understanding of the *Cx*. *pipiens* complex we have produced may inform those examining these mosquitoes as agents of disease transmission.

## Supplementary information

**Additional file 1: Table S1.** Sample information for the data used in this study. **Table S2.** Results from our ADMIXTURE analysis showing the proportion of genetic diversity derived from each cluster for the ‘four-fold degenerate segregating sites’ dataset. **Table S3.** Results from our ADMIXTURE analysis showing the proportion of genetic diversity derived from each cluster for the ‘all segregating sites’ dataset. **Table S4.** The raw cross-validation (CV) error values from our ADMIXTURE analyses for both the ‘four-fold degenerate sites’ and ‘all segregating sites’ dataset analyses. **Table S5.** The results from the Structure Harvester analysis of our STRUCTURE results. **Table S6.** The posterior probability of each of five replicates for each K value in our structure analyses. **Table S7.** Pairwise unweighted and weighted F_st_ values for each of five taxonomic clusters.

**Additional file 2: Figure S1.** The observed distributions of variant quality measures in our ‘all segregating sites’ dataset. **Figure S2. a** The number of segregating variants in our ‘four-fold degenerate sites’ dataset across the three *Culex* chromosomes. **b** The number of segregating variants in our ‘all segregating sites’ dataset across the three *Culex* chromosomes. **Figure S3.** Principal components analysis (PCA) using all segregating sites with reported samples for all six described members of the *Culex pipiens* complex (**a**) and with a four-taxon set that excluded *australicus* and *globocoxitus* (**b**). **Figure S4.** Violin plots showing the cross-validation (CV) error values from our ADMIXTURE analyses. **Figure S5.** World maps showing the relative proportions of inferred populations as determined in our ADMIXTURE analysis using four-fold degenerate sites. **Figure S6.** World maps showing the relative proportions of inferred populations as determined in our ADMIXTURE analysis using all segregating sites. **Figure S7.** STRUCTURE bar plots for the samples in our subsampled dataset using all segregating sites. **Figure S8.** Maximum likelihood phylogeny using all segregating sites.

## Data Availability

Previously unpublished data are available in the National Center for Biotechnology Information’s Short Read Archive database (NCBI-SRA), under accession numbers SRR10053379-SRR10053386 (BioProject: PRJNA561911).

## Abbreviations

GATK: Genome Analysis Toolkit
SNP: single nucleotide polymorphism
QD: quality by depth
FS: Fisher strand bias
MQ: mapping quality
MQRankSum: mapping quality rank sum
ReadPosRankSum: read position rank sum
SOR: strand odds ratio
PCA: principal component analysis
PC: principal component
K: genetic cluster
ML: maximum likelihood
TVM: transversional model
GTR: generalized time reversible model
AIC: Akaike information criterion
Fst: fixation Index
CV: cross-validation
NCA: North and Central America
EMD: Europe and the Mediterranean
SSA: sub-Saharan Africa
CSA: China and Southeast Asia
AUS: Australia

## References

[1] Hey J, Waples RS, Arnold ML, Butlin RK, Harrison RG (2003). Understanding and confronting species uncertainty in biology and conservation. Trends Ecol Evol..

[2] Degnan JH, Rosenberg NA (2009). Gene tree discordance, phylogenetic inference and the multispecies coalescent. Trends Ecol Evol..

[3] Arnold ML (2015). Divergence with genetic exchange.

[4] Fontaine MC, Pease JB, Steele A, Waterhouse RM, Neafsey DE, Sharakhov IV (2015). Mosquito genomics. Extensive introgression in a malaria vector species complex revealed by phylogenomics. Science..

[5] Smith JL, Fonseca DM (2004). Rapid assays for identification of members of the *Culex* (*Culex*) *pipiens* complex, their hybrids, and other sibling species (Diptera: Culicidae). Am J Trop Med Hyg..

[6] Harbach RE (2012). *Culex pipiens*: species *versus* species complex—taxonomic history and perspective. J Am Mosq Control Assoc..

[7] Russell RC (2012). A review of the status and significance of the species within the *Culex pipiens* group in Australia. J AM Mosq Control Assoc..

[8] Paterson HE, James SH (1973). Animal and plant speciation studies in western Australia. J R Soc West Aust..

[9] Batovska J, Blacket MJ, Brown K, Lynch SE (2016). Molecular identification of mosquitoes (Diptera: Culicidae) in southeastern Australia. Ecol Evol..

[10] Dobrotworsky NV. The *Culex pipiens* group in south-eastern Australia I. In: Proc Linn Soc New South Wales, vol. 77. 1952. p. 357–60.

[11] Dobrotworsky NV. The *Culex pipiens* group in south-eastern Australia II. In: Proc Linn Soc New South Wales, vol. 78. 1953. p. 131–46.

[12] Dobrotworsky NV. The *Culex pipiens* group in south-eastern Australia IV. Crossbreeding experiments within the *Culex pipiens* group. In: Proc Linn Soc New South Wales, vol. 80. 1955. p. 33–43.

[13] Dobrotworsky NV (1967). The problem of the *Culex pipiens* complex in the South Pacific (including Australia). Bull World Health Organ..

[14] Vinogradova EB (2000). *Culex pipiens pipiens* mosquitoes: taxonomy, distribution, ecology, physiology, genetics, applied importance and control.

[15] Miles SJ, Paterson HE (1979). Protein variation and systematics in the *Culex pipiens* group of species. Mosq Syst..

[16] Barr AR (1967). Occurrence and distribution of the *Culex pipiens* complex. Bull World Health Organ..

[17] Cornel AJ, McAbee RD, Rasgon J, Stanich MA, Scott TW, Coetzee M (2003). Differences in extent of genetic introgression between sympatric *Culex pipiens* and *Culex quinquefasciatus* (Diptera: Culicidae) in California and South Africa. J Med Entomol..

[18] Tanaka K, Mizusawa K, Saugstad ES (1979). A revision of the adult and larval mosquitoes of Japan (including the Ryukyu Archipelago and the Ogasawara Islands) and Korea (Diptera: Culicidae). Contrib Amer Ent Inst..

[19] Sasa M, Shirasaka A, Kurihara T (1966). Crossing experiments between *fatigans*, *pallens* and *molestus* colonies of the mosquito *Culex pipiens s.1.* from Japan and Southern Asia, with special reference to hatchability of hybrid eggs. Jpn J Exp Med..

[20] Hubert AA, Young JL, Kato A (1971). Genetic incompatibility and hybridization studies on two members of the *Culex pipiens* complex. J Med Entomol..

[21] Mogi M (2012). The forms of the *Culex pipiens* complex in East Asia, with ecological thoughts on their origin and interrelation. J Am Mosq Control Assoc..

[22] Ohashi K, Tsuda Y, Kasai S, Kawada H, Takagi M (2014). Hybridization between sympatric populations of *Culex pipiens pallens* and *Culex pipiens* f. *molestus* (Diptera: Culicidae) in Nagasaki, Japan. Med Entomol Zool..

[23] Wu T, Hu Q, Zhao T, Tian J, Xue R (2014). Morphological studies on *Culex molestus* of the *Culex pipiens* complex (Diptera: Culicidae) in underground parking lots in Wuhan, central China. Fla Entomol..

[24] Liu L, Zhang B, Cheng P, Wang H, Guo X, Zhang C (2016). Overwintering of *Culex pipiens pallens* (Diptera: Culicidae) in Shandong, China. J. Entomol Sci..

[25] Fonseca DM, Smith JL, Kim HC, Mogi M (2009). Population genetics of the mosquito *Culex pipiens pallens* reveals sex-linked asymmetric introgression by *Culex quinquefasciatus*. Infect Genet Evol..

[26] Richards AG (1941). A stenogamic autogenous strain of *Culex pipiens* L. in North America (Diptera: Culicidae). Entomol News.

[27] Shaikevich EV (2007). PCR-RFLP of the COI gene reliably differentiates *Cx. pipiens*, *Cx. pipiens* f. *molestus* and *Cx. torrentium* of the *pipiens* complex. Eur Mosq Bull..

[28] Kothera L, Godsey M, Mutebi JP, Savage HM (2010). A comparison of aboveground and belowground populations of *Culex pipiens* (Diptera: Culicidae) mosquitoes in Chicago, Illinois, and New York City, New York, using microsatellites. J Med Entomol..

[29] Kothera L, Godsey M, Mutebi JP, Savage HM (2012). A comparison of above-ground and below-ground populations of *Culex pipiens pipiens* in Chicago, Illinois, and New York City, New York, using 2 microsatellite assays. J Am Mosq Control Assoc..

[30] Kothera L, Nelms B, Savage HM, Reisen WK. Complexity of the *Culex pipiens* complex in California. In: Proc Pap Annu Conf Mosq Vector Control Assoc Calif, vol. 80. 2012. p. 1–3. PMC5502828 28701905

[31] Kothera L, Nelms BM, Reisen WK, Savage HM (2013). Population genetic and admixture analyses of *Culex pipiens* complex (Diptera: Culicidae) populations in California, United States. Am J Trop Med Hyg..

[32] Fonseca DM, Keyghobadi N, Malcolm CA, Mehmet C, Schaffner F, Mogi M (2004). Emerging vectors in the *Culex pipiens* complex. Science..

[33] Bahnck CM, Fonseca DM (2006). Rapid assay to identify the two genetic forms of *Culex* (*Culex*) *pipiens* L. (Diptera: Culicidae) and hybrid populations. Am J Trop Med Hyg..

[34] Strickman D, Fonseca DM (2012). Autogeny in *Culex pipiens* complex mosquitoes from the San Francisco Bay Area. Am J Trop Med Hyg..

[35] Farajollahi A, Fonseca DM, Kramer LD, Kilpatrick AM (2011). “Bird biting” mosquitoes and human disease: a review of the role of *Culex pipiens* complex mosquitoes in epidemiology. Infect Genet Evol..

[36] Micieli MV, Matacchiero AC, Muttis E, Fonseca DM, Aliota MT, Kramer LD (2013). Vector competence of Argentine mosquitoes (Diptera: Culicidae) for West Nile virus (Flaviviridae: Flavivirus). J Med Entomol..

[37] Vogels CBF, Fros JJ, Göertz GP, Pijlman GP, Koenraadt CJM (2016). Vector competence of northern European *Culex pipiens* biotypes and hybrids for West Nile virus is differentially affected by temperature. Parasites Vectors..

[38] Shi M, Neville P, Nicholson J, Eden JS, Imrie A, Holmes EC (2017). High-resolution metatranscriptomics reveals the ecological dynamics of mosquito-associated RNA viruses in western Australia. J Virol..

[39] Romiguier J, Lourenco J, Gayral P, Faivre N, Weinert LA, Ravel S (2014). Population genomics of eusocial insects: the costs of a vertebrate-like effective population size. J Evol Biol..

[40] Li CX, Guo XX, Zhang YM, Dong YD, Xing D, Yan T (2016). Identification of genes involved in pyrethroid-, propoxur-, and dichlorvos- insecticides resistance in the mosquitoes, *Culex pipiens* complex (Diptera: Culicidae). Acta Trop..

[41] Honnen AC, Johnston PR, Monaghan MT (2016). Sex-specific gene expression in the mosquito *Culex pipiens* f. *molestus* in response to artificial light at night. BMC Genomics..

[42] Asgharian H, Chang PL, Lysenkov S, Scobeyeva VA, Reisen WK, Nuzhdin SV (2015). Evolutionary genomics of *Culex pipiens*: global and local adaptations associated with climate, life-history traits and anthropogenic factors. Proc Biol Sci..

[43] Price DC, Fonseca DM (2015). Genetic divergence between populations of feral and domestic forms of a mosquito disease vector assessed by transcriptomics. PeerJ..

[44] Bigot D, Atyame CM, Weill M, Justy F, Herniou EA, Gayral P (2018). Discovery of *Culex pipiens* associated Tunisia virus: a new ssRNA(+) virus representing a new insect associated virus family. Virus Evol..

[45] Chandler JA, Liu RM, Bennett SN (2015). RNA shotgun metagenomic sequencing of northern California (USA) mosquitoes uncovers viruses, bacteria, and fungi. Front Microbiol..

[46] Hagan RW, Didion EM, Rosselot AE, Holmes CJ, Siler SC, Rosendale AJ (2018). Dehydration prompts increased activity and blood feeding by mosquitoes. Sci Rep..

[47] Leal WS, Choo YM, Xu P, da Silva CS, Ueira-Vieira C (2013). Differential expression of olfactory genes in the southern house mosquito and insights into unique odorant receptor gene isoforms. Proc Natl Acad Sci USA.

[48] Reid WR, Zhang L, Liu F, Liu N (2012). The transcriptome profile of the mosquito *Culex quinquefasciatus* following permethrin selection. PLoS ONE.

[49] Arensburger P, Megy K, Waterhouse RM, Abrudan J, Amedeo P, Antelo B (2010). Sequencing of *Culex quinquefasciatus* establishes a platform for mosquito comparative genomics. Science..

[50] Cutler DJ, Jensen JD (2010). To pool, or not to pool?. Genetics..

[51] Anderson EC, Skaug HJ, Barshis DJ (2014). Next-generation sequencing for molecular ecology: a caveat regarding pooled samples. Mol Ecol..

[52] Ellegren H (2014). Genome sequencing and population genomics in non-model organisms. Trends Ecol Evol..

[53] Mutebi JP, Savage HM (2009). Discovery of *Culex pipiens pipiens* form *molestus* in Chicago. J Am Mosq Control Assoc..

[54] Dudchenko O, Batra SS, Omer AD, Nyquist SK, Hoeger M, Durand NC (2017). *De novo* assembly of the *Aedes aegypti* genome using Hi-C yields chromosome-length scaffolds. Science..

[55] Dobin A, Davis CA, Schlesinger F, Drenkow J, Zaleski C, Jha S (2013). STAR: ultrafast universal RNA-seq aligner. Bioinformatics..

[56] Dobin A, Gingeras TR (2015). Mapping RNA-seq reads with STAR. Curr Protoc Bioinformatics..

[57] Li H. Aligning sequence reads, clone sequences and assembly contigs with BWA-MEM. 2013; v2 [q-bio.GN]. rXiv preprint, arXiv:1303.3997.

[58] McKenna A, Hanna M, Banks E, Sivachenko A, Cibulskis K, Kernytsky A (2010). The genome analysis toolkit: a MapReduce framework for analyzing next-generation DNA sequencing data. Genome Res..

[59] Hershberg R, Petrov DA (2008). Selection on codon bias. Annu Rev Genet..

[60] Sella G, Petrov DA, Przeworski M, Andolfatto P (2009). Pervasive natural selection in the *Drosophila* genome?. PLoS Genet..

[61] Lawrie DS, Messer PW, Hershberg R, Petrov DA (2013). Strong purifying selection at synonymous sites in *D. melanogaster*. PLoS Genet..

[62] Hoff KJ, Lomsadze A, Borodovsky M, Stanke M (2019). Whole-genome annotation with BRAKER. Methods Mol Biol..

[63] Cingolani P, Platts A, le Wang L, Coon M, Nguyen T, Wang L (2012). A program for annotating and predicting the effects of single nucleotide polymorphisms, SnpEff: SNPs in the genome of *Drosophila melanogaster* strain w1118; iso-2; iso-3. Fly.

[64] Li H (2011). A statistical framework for SNP calling, mutation discovery, association mapping and population genetical parameter estimation from sequencing data. Bioinformatics..

[65] Danecek P, Auton A, Abecasis G, Albers CA, Banks E, DePristo MA (2011). The variant call format and VCFtools. Bioinformatics..

[66] Pritchard JK, Stephens M, Donnelly P (2000). Inference of population structure using multilocus genotype data. Genetics..

[67] Purcell S, Neale B, Todd-Brown K, Thomas L, Ferreira MAR, Bender D (2007). PLINK: a toolset for whole-genome association and population-based linkage analysis. Am J Hum Genet..

[68] Novembre J, Johnson T, Bryc K, Kutalik Z, Boyko AR, Auton A (2008). Genes mirror geography within Europe. Nature..

[69] Kofler R, Orozco-terWengel P, De Maio N, Pandey RV, Nolte V, Futschik A (2011). PoPoolation: a toolbox for population genetic analysis of next generation sequencing data from pooled individuals. PLoS ONE..

[70] Ferretti L, Ramos-Onsins SE, Pérez-Enciso M (2013). Population genomics from pool sequencing. Mol Ecol..

[71] Hivert V, Leblois R, Petit EJ, Gautier M, Vitalis R (2018). Measuring genetic differentiation from pool-seq data. Genetics..

[72] Weir BS, Cockerham CC (1984). Estimating F-statistics for the analysis of population structure. Evolution..

[73] Weir BS, Hill WG (2002). Estimating F-statistics. Annu Rev Genet..

[74] R Core Team. R: A language and environment for statistical computing. Vienna: R Foundation for Statistical Computing; 2018. https://www.R-project.org/. Accessed 2 Jul 2018.

[75] Alexander DH, Novembre J, Lange K (2009). Fast model-based estimation of ancestry in unrelated individuals. Genome Res..

[76] Alexander DH, Shringarpure SS, Novembre J, Lange K (2015). Admixture 1.3 software manual.

[77] Kopelman NM, Mayzel J, Jakobsson M, Rosenberg NA, Mayrose I (2015). Clumpak: a program for identifying clustering modes and packaging population structure inferences across K. Mol Ecol Resour..

[78] Schwartz MK, McKelvey KS (2009). Why sampling scheme matters: the effect of sampling scheme on landscape genetic results. Conserv Genet..

[79] Puechmaille SJ (2016). The program STRUCTURE does not reliably recover the correct population structure when sampling is uneven: subsampling and new estimators alleviate the problem. Mol Ecol Resour..

[80] Meirmans PG (2019). Subsampling reveals that unbalanced sampling affects STRUCTURE results in a multi-species dataset. Heredity..

[81] Earl DA, vonHoldt BM (2012). STRUCTURE HARVESTER: a website and program for visualizing STRUCTURE output and implementing the Evanno method. Conserv Genet Resour..

[82] Evanno G, Regnaut S, Goudet J (2005). Detecting the number of clusters of individuals using the software STRUCTURE: a simulation study. Mol Ecol..

[83] Pritchard JK, Wen W, Falush D (2010). Documentation for structure software: version 2.3.

[84] Posada D, Baxevanis AD, Davison DB, Page RDM, Petsko GA, Stein LD, Stormo GD (2003). Using Modeltest and PAUP* to select a model of nucleotide substitution. Current protocols in bioinformatics.

[85] Tavaré S (1986). Some probabilistic and statistical problems in the analysis of DNA sequences. Lect Math Life Sci..

[86] Guindon S, Gascuel O (2003). A simple, fast and accurate method to estimate large phylogenies by maximum-likelihood. Syst Biol..

[87] Darriba D, Taboada GL, Doallo R, Posada D (2012). jModelTest 2: more models, new heuristics and parallel computing. Nat Methods..

[88] Guindon S, Dufayard JF, Lefort V, Anisimova M, Hordijk W, Gascuel O (2010). New algorithms and methods to estimate maximum-likelihood phylogenies: assessing the performance of PhyML 3.0. Syst Biol..

[89] Stamatakis A (2014). RAxML version 8: a tool for phylogenetic analysis and post-analysis of large phylogenies. Bioinformatics..

[90] Fitzpatrick BM (2012). Estimating ancestry and heterozygosity of hybrids using molecular markers. BMC Evol Biol..

[91] Cornel A, Lee Y, Fryxell RT, Siefert S, Nieman C, Lanzaro G (2012). *Culex pipiens sensu lato* in California: a complex within a complex?. J Am Mosq Control Assoc..

[92] Harbach RE, Harrison BA, Gad AM (1984). *Culex* (*Culex*) *molestus* Forskål (Diptera: Culicidae): neotype designation, description, variation, and taxonomic status. Proc Entomol Soc Wash..

[93] Gillespie JH (2004). Population genetics: a concise guide.

