# Supplementary material for: Global evaluation of taxonomic relationships and admixture within the Culex pipiens complex of mosquitoes
Source: Parasit Vectors. 2020 Jan 8;13:8. doi: 10.1186/s13071-020-3879-8 (PMC6950815; doi:10.1186/s13071-020-3879-8)
Supplement: Supplementary file 2 — Additional file 2: Figure S1. The observed distributions of variant quality measures in our ‘all segregating sites’ dataset. Figure S2. a The number of segregating variants in our ‘four-fold degenerate sites’ dataset across the three Culex chromosomes. b The number of segregating variants in our ‘all segregating sites’ dataset across the three Culex chromosomes. Figure S3. Principal components analysis (PCA) using all segregating sites with reported samples for all six described members of the Culex pipiens complex (a) and with a four-taxon set that excluded australicus and globocoxitus (b). Figure S4. Violin plots showing the cross-validation (CV) error values from our ADMIXTURE analyses. Figure S5. World maps showing the relative proportions of inferred populations as determined in our ADMIXTURE analysis using four-fold degenerate sites. Figure S6. World maps showing the relative proportions of inferred populations as determined in our ADMIXTURE analysis using all segregating sites. Figure S7. STRUCTURE bar plots for the samples in our subsampled dataset using all segregating sites. Figure S8. Maximum likelihood phylogeny using all segregating sites. [file 13071_2020_3879_MOESM2_ESM.pdf]

Additional file 2

Figure S1. The observed distributions of variant quality measures in our 'all segregating sites' dataset. The distributions in our 'four-fold degenerate sites' dataset were nearly identical (not shown). The red lines indicate the filtering thresholds chosen. SNPs that had a quality by depth less than 2 (QD < 2.0), Fisher strand bias greater than 40 (FS > 40.0), mapping quality less than 55 (MQ < 55.0), mapping quality rank sum less than -0.2 (MQRankSum < -0.2), read position rank sum less than -2 (ReadPosRankSum < -2.0), and/or a strand odds ratio greater than 3 (SOR > 3.0), were removed from our datasets.

Figure S2. a The number of segregating variants in our 'four-fold degenerate sites' dataset within 10 kb windows across the three *Culex* chromosomes. **b** The number of segregating variants in our 'all segregating sites' dataset within 10 kb windows across the three *Culex* chromosomes. In both **a** and **b**, the orange arrows along each chromosome represent the approximate location of the centromere.

Figure S3. Principal components analysis (PCA) using all segregating sites with reported samples for all six described members of the *Culex pipiens* complex (**a**) and with a four-taxon set that excluded the reported Australian endemic taxa, *australicus* and *globocoxitus* (**b**). These PCAs were implemented with PLINK and plotted in R. Shown are the first two PCs. Colors corresponding to the different designated taxa are consistent between the two PCAs.

Figure S4. Violin plots showing the cross-validation (CV) error values from our ADMIXTURE analyses using four-fold degenerate sites (top) and all segregating sites (bottom). Shown are results utilizing K values from 1 to 7. Mean values are represented by the red dots. The K value with the lowest CV score was considered to be the best-supported number of clusters. Each K value was run 20 independent times.

Figure S5. World maps showing the relative proportions of inferred populations as determined in our ADMIXTURE analysis (large circles) for K = 2–7, using four-fold degenerate sites. Note that for our sample designations, we defined five broad geographical regions, indicated on the map by the dashed gray boxes. See Table 1 and S1 for more information on the samples.

Figure S6. World maps showing the relative proportions of inferred populations as determined in our ADMIXTURE analysis (large circles) for K = 2–7, using all segregating sites. Note that for our sample designations, we defined five broad geographical regions, indicated on the map by the dashed gray boxes. See Table 1 and Additional file 1: Table S1 for more information on the samples.

Figure S7. Maximum likelihood phylogenic analysis using all segregating sites and a generalized time reversible model of nucleotide substitution with a gamma distribution of rate heterogeneity (GTR + Γ [80]). The colors for the branch tip labels correspond to the six different taxa in this study. The numbers at the major branch nodes indicate bootstrap support for each bifurcation in the tree (out of 100). The three-letter code in the middle of each sample name indicates its geographical region of origin. For additional sample details, see Additional file 1: Table S1. Samples under a broad dashed line were determined to be intra-taxonomically admixed (*pipiens* and *molestus* only). Samples under a fine dashed line were determined to be inter-taxonomically admixed. Within the *pipiens* and *molestus* samples, three broad geographical clusters are defined: North America, Mediterranean and northern Europe (including Russia).

Figure S8. STRUCTURE bar plots for the samples in our subsampled dataset using all segregating sites, plotted for genetic clusters (K) from two through seven. For each horizontal bar, the relative proportions of each color indicate the proportion of genetic diversity assigned to that cluster. Sample designations are reported along the left y-axis. Designated taxon groups are reported along the right y-axis. For additional sample details, see Table 1 and Additional file 1: Table S1.

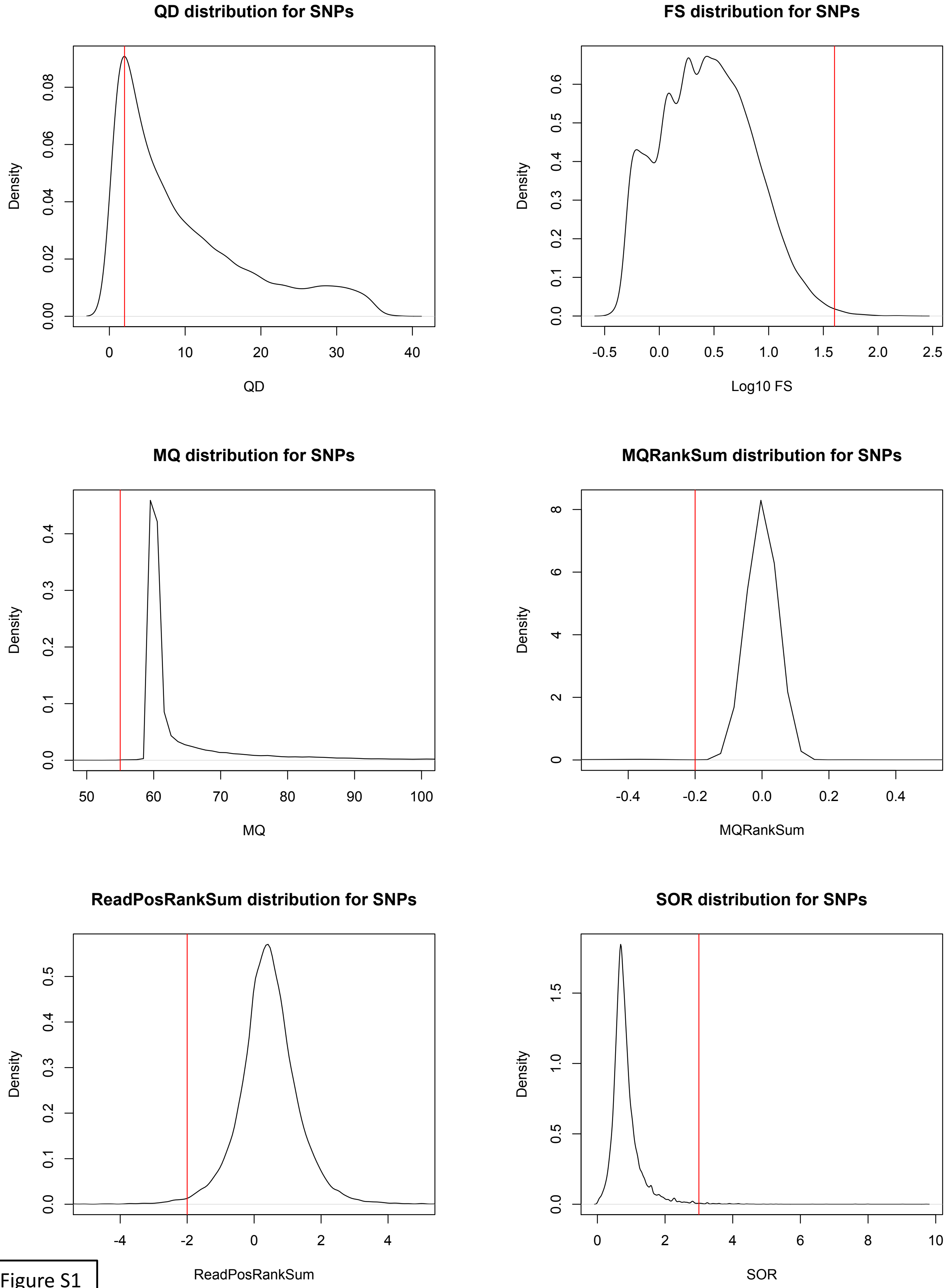
QD distribution for SNPs
Density
QD
FS distribution for SNPs
Density
Log10 FS
MQ distribution for SNPs
Density
MQ
MQRankSum distribution for SNPs
Density
MQRankSum
ReadPosRankSum distribution for SNPs
Density
ReadPosRankSum
SOR distribution for SNPs
Density
SOR

Figure S1

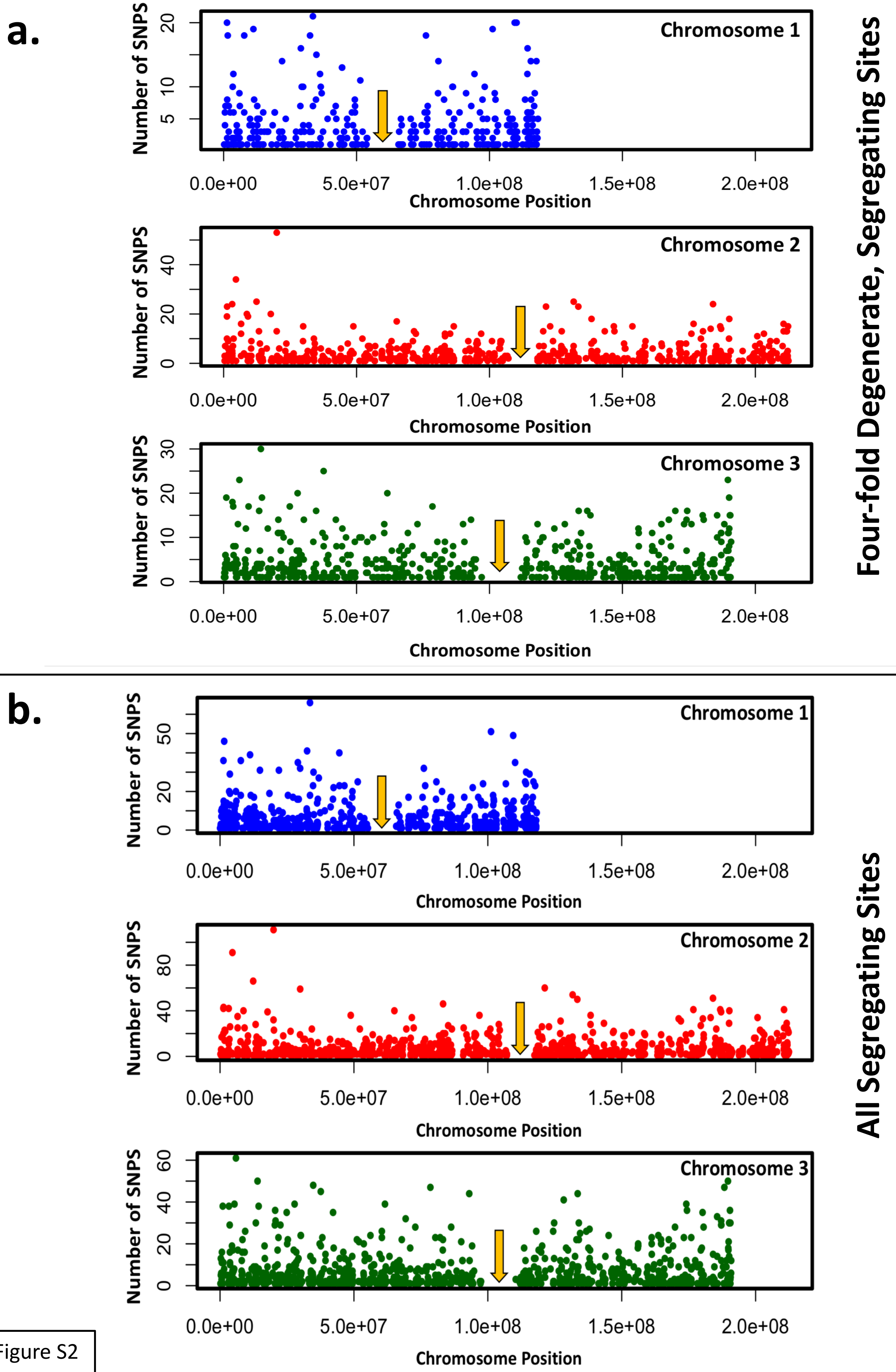
a.
Four-fold Degenerate, Segregating Sites
Chromosome 1
Chromosome 2
Chromosome 3
Number of SNPS
Chromosome Position
0.0e+00
5.0e+07
1.0e+08
1.5e+08
2.0e+08
b.
All Segregating Sites
Chromosome 1
Chromosome 2
Chromosome 3
Number of SNPS
Chromosome Position

Figure S2

Figure S3

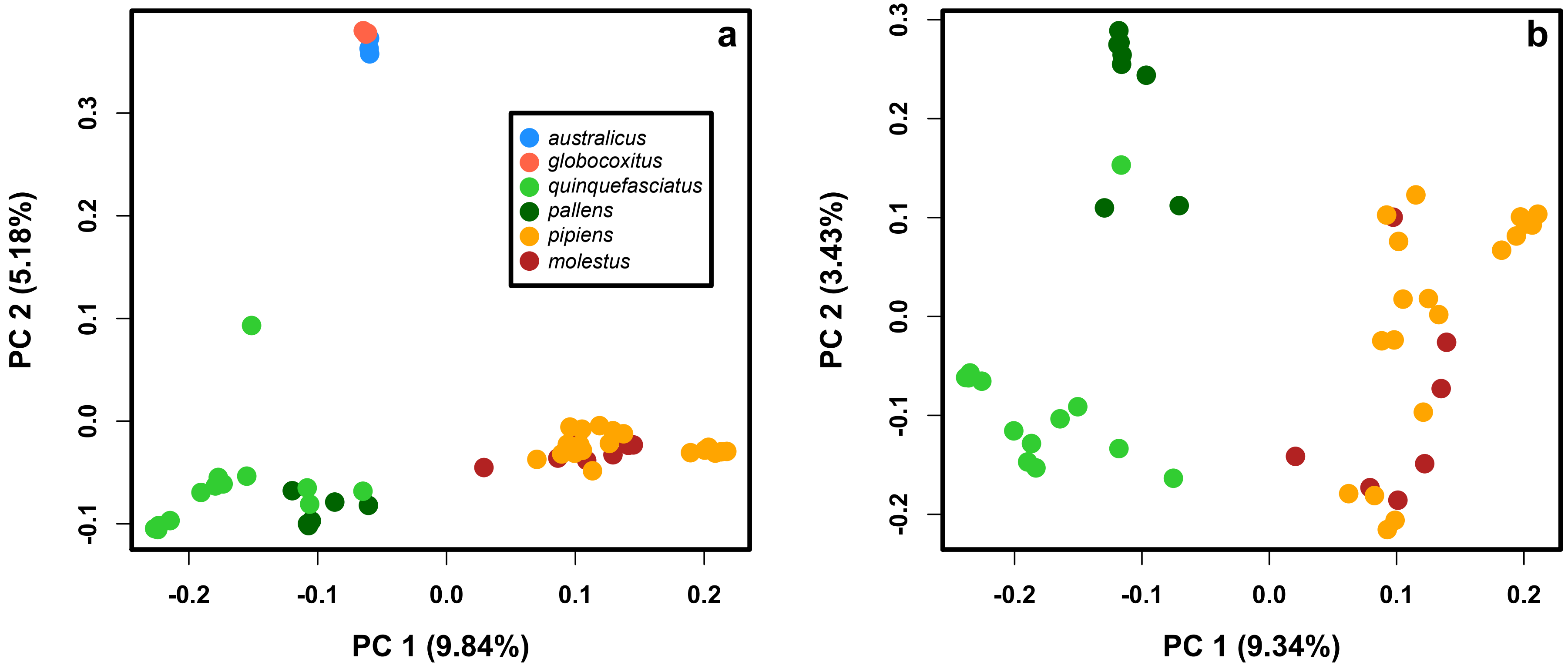
a
australicus
globocoxitus
quinquefasciatus
pallens
pipiens
molestus
PC 2 (5.18%)
PC 1 (9.84%)
b
PC 2 (3.43%)
PC 1 (9.34%)

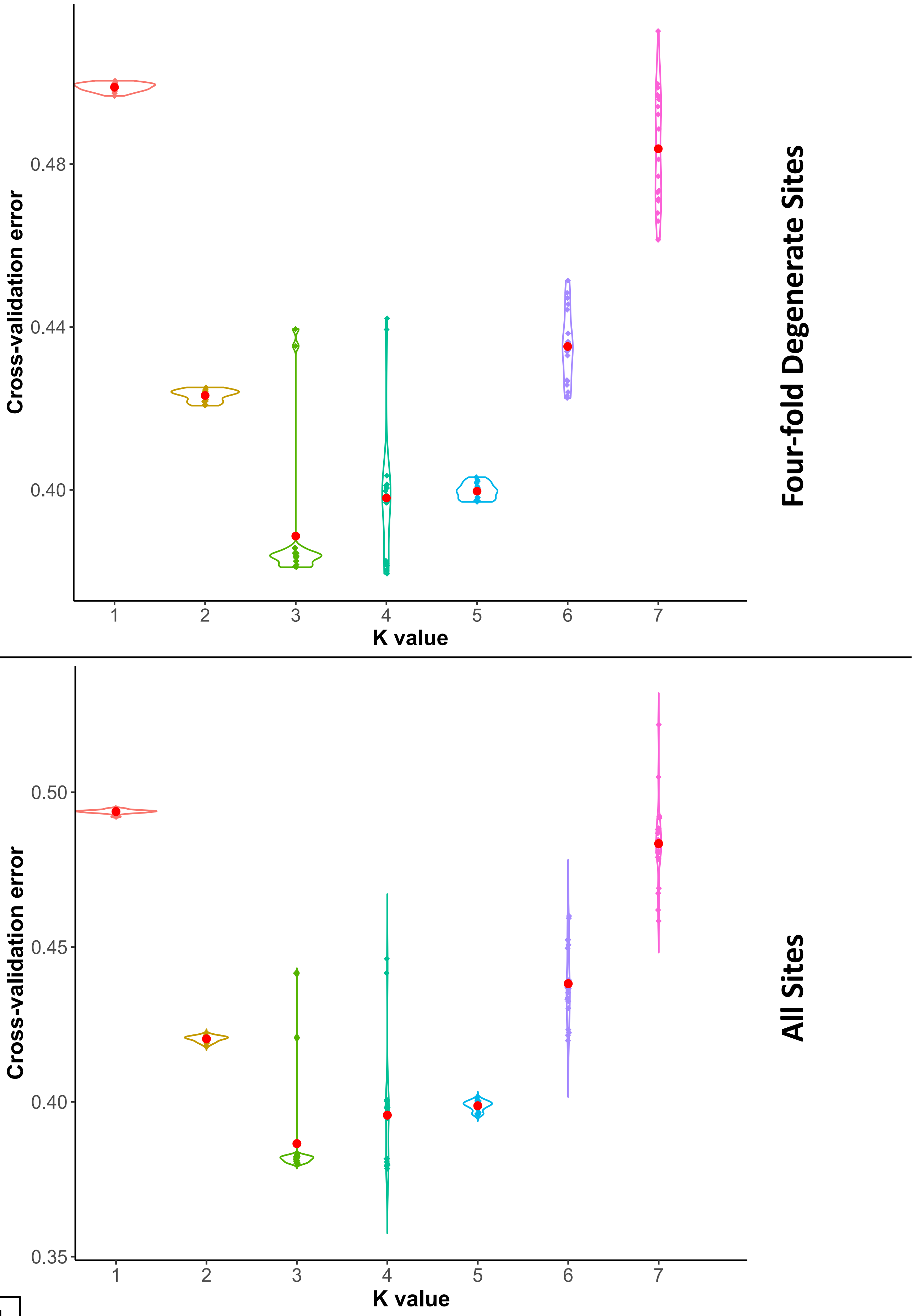

Four-fold Degenerate Sites
Cross-validation error
0.48
0.44
0.40
K value
1
2
3
4
5
6
7
All Sites
Cross-validation error
0.50
0.45
0.40
0.35
K value
1
2
3
4
5
6
7

Figure S4

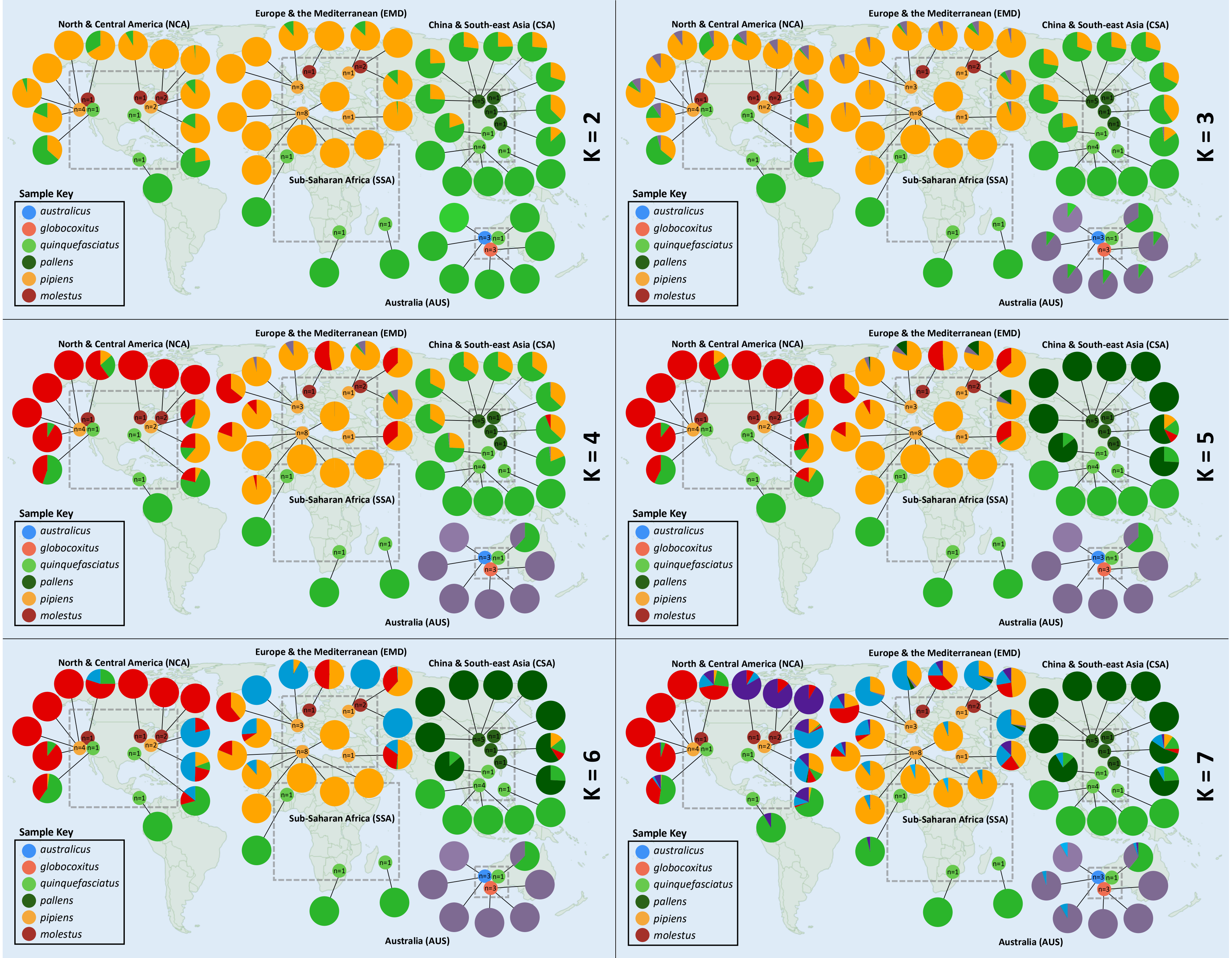
K = 2
K = 3
K = 4
K = 5
K = 6
K = 7
North & Central America (NCA)
Europe & the Mediterranean (EMD)
China & South-east Asia (CSA)
Sub-Saharan Africa (SSA)
Australia (AUS)
Sample Key
australicus
globocoxitus
quinquefasciatus
pallens
pipiens
molestus

Figure S5

Figure S6

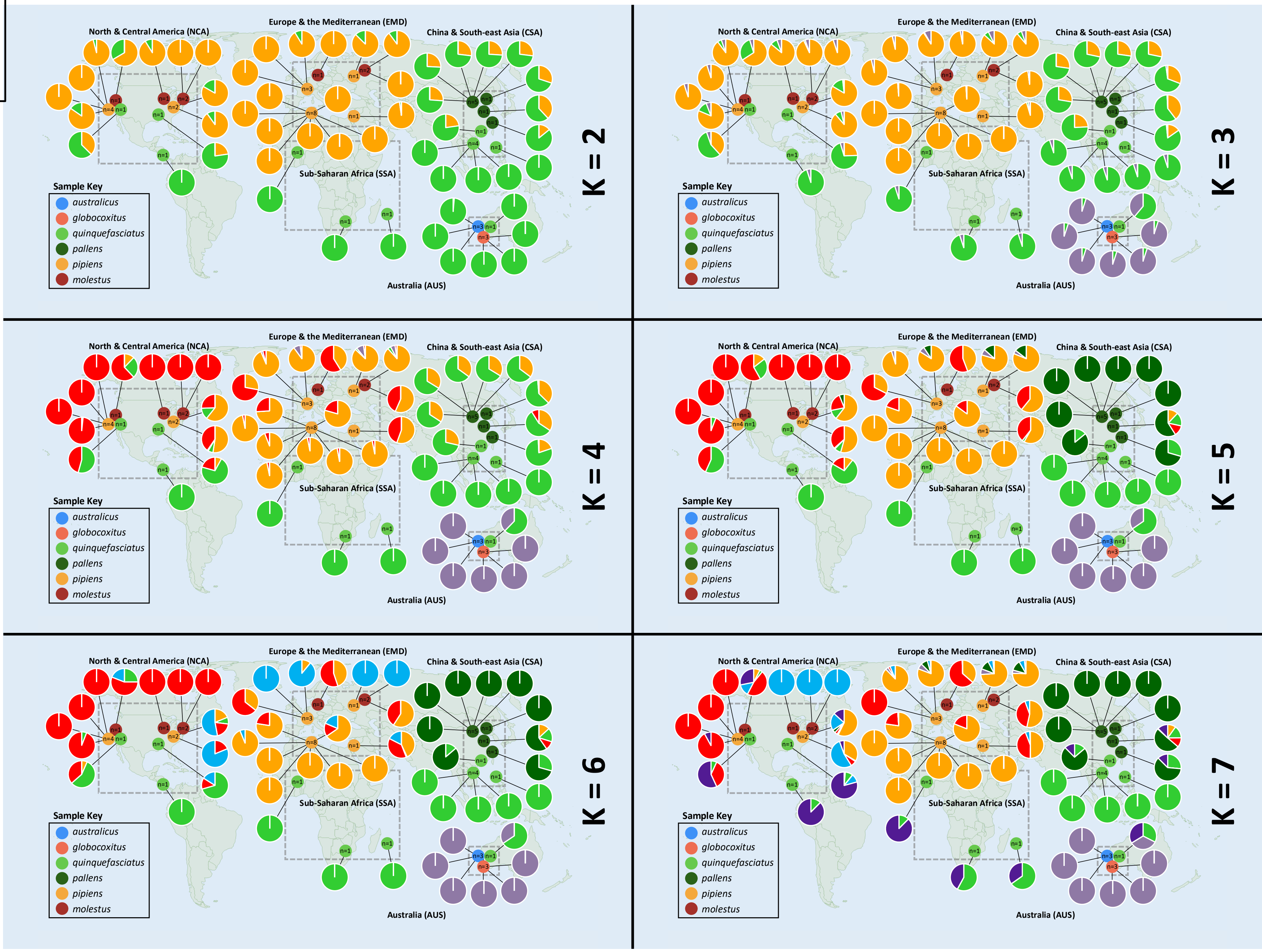
K = 2
K = 3
K = 4
K = 5
K = 6
K = 7
North & Central America (NCA)
Europe & the Mediterranean (EMD)
China & South-east Asia (CSA)
Sub-Saharan Africa (SSA)
Australia (AUS)
Sample Key
australicus
globocoxitus
quinquefasciatus
pallens
pipiens
molestus

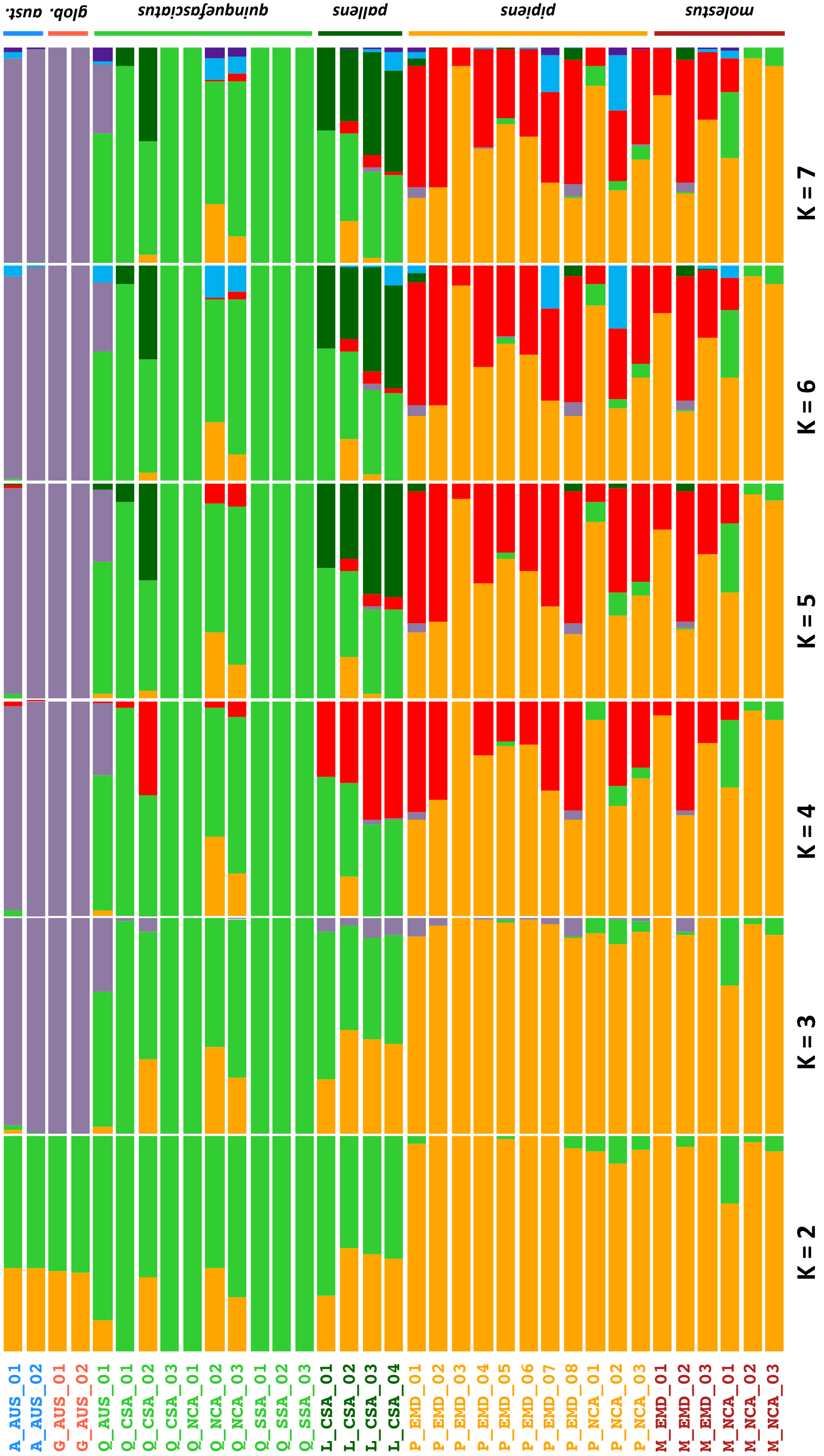

aust.
glob.
quinquefasciatus
pallens
pipiens
molestus
K = 2
K = 3
K = 4
K = 5
K = 6
K = 7
A_AUS_01
A_AUS_02
G_AUS_01
G_AUS_02
Q_AUS_01
Q_CSA_01
Q_CSA_02
Q_CSA_03
Q_NCA_01
Q_NCA_02
Q_NCA_03
Q_SSA_01
Q_SSA_02
Q_SSA_03
L_CSA_01
L_CSA_02
L_CSA_03
L_CSA_04
P_EMD_01
P_EMD_02
P_EMD_03
P_EMD_04
P_EMD_05
P_EMD_06
P_EMD_07
P_EMD_08
P_NCA_01
P_NCA_02
P_NCA_03
M_EMD_01
M_EMD_02
M_EMD_03
M_NCA_01
M_NCA_02
M_NCA_03

Figure S7

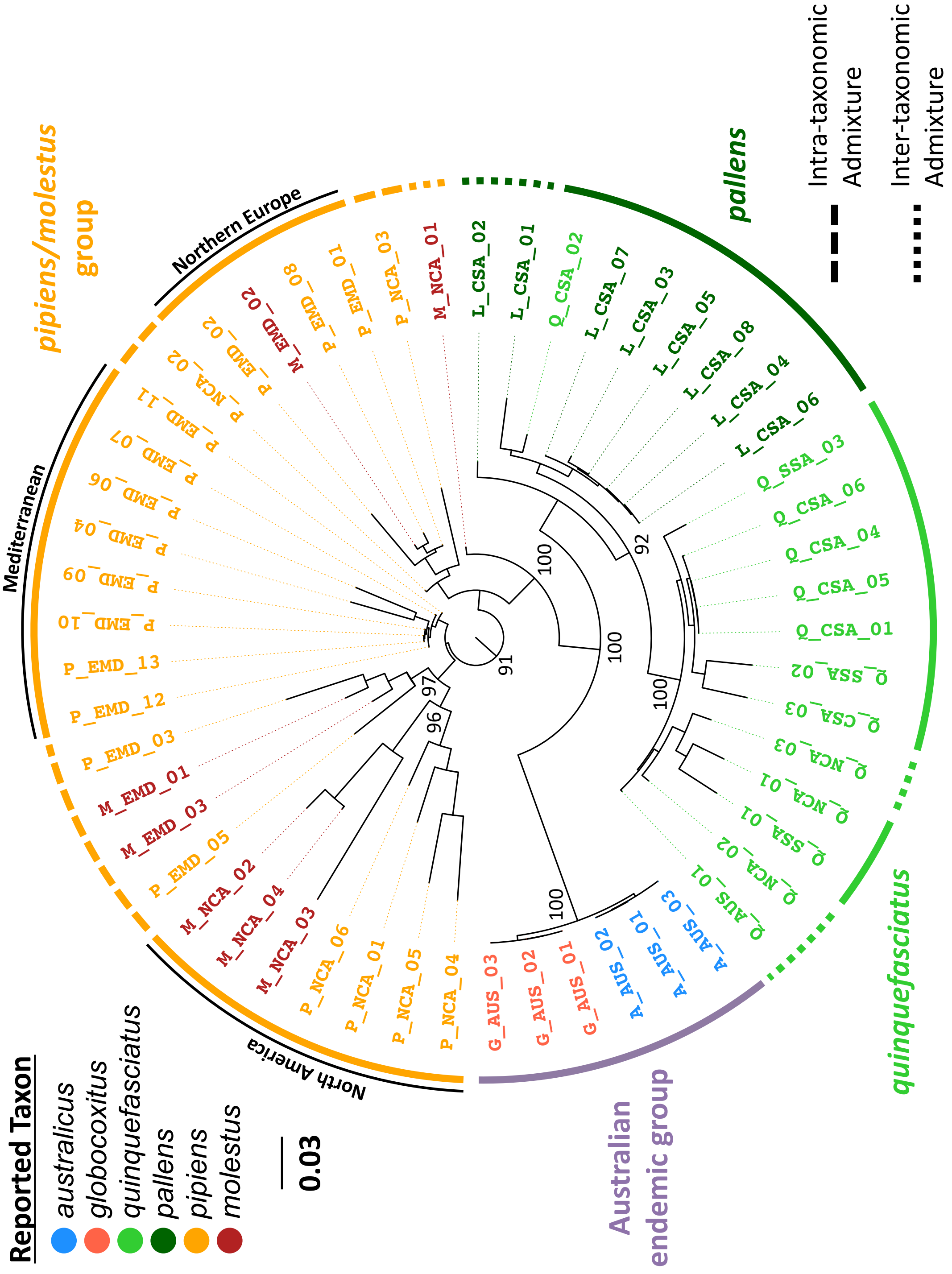

pipiens/molestus group
pallens
quinquefasciatus
Australian endemic group
Northern Europe
Mediterranean
North America
Intra-taxonomic Admixture
Inter-taxonomic Admixture
Reported Taxon
australicus
globocoxitus
quinquefasciatus
pallens
pipiens
molestus
0.03
M_NCA_01
P_NCA_03
P_EMD_01
P_EMD_08
M_EMD_02
P_EMD_02
P_NCA_02
P_EMD_11
P_EMD_07
P_EMD_06
P_EMD_04
P_EMD_09
P_EMD_10
P_EMD_13
P_EMD_12
P_EMD_03
M_EMD_01
M_EMD_03
P_EMD_05
M_NCA_02
M_NCA_04
M_NCA_03
P_NCA_06
P_NCA_01
P_NCA_05
P_NCA_04
G_AUS_03
G_AUS_02
G_AUS_01
A_AUS_02
A_AUS_01
A_AUS_03
Q_AUS_01
Q_NCA_02
Q_SSA_01
Q_NCA_01
Q_NCA_03
Q_CSA_03
Q_SSA_02
Q_CSA_01
Q_CSA_05
Q_CSA_04
Q_CSA_06
Q_SSA_03
L_CSA_06
L_CSA_04
L_CSA_08
L_CSA_05
L_CSA_03
L_CSA_07
Q_CSA_02
L_CSA_01
L_CSA_02
100
92
100
100
91
97
96
100

Figure S8
